# Functionalization‐Mediated Synthesis of Bridge‐Coordinated Fe Atoms on MoS_2_ Nanosheets for PEM Water Electrolyzers

**DOI:** 10.1002/anie.6960943

**Published:** 2026-07-27

**Authors:** Zakaria Anfar, Haitham Maslouh, Yuwei Yang, Mathilde Moderne, Jiefeng Liu, Loïc Assaud, Mikhael Bechelany, Chrystelle Salameh, Kun Qi, Yuying Meng, Huali Wu, Ji Li, Nicholas M. Bedford, Damien Voiry

**Affiliations:** ^1^ Institut Européen Des Membranes (IEM), CNRS, ENSCM University of Montpellier (UM) Montpellier France; ^2^ Institut de Chimie Moléculaire Et Des Matériaux d'Orsay (ICMMO) Université Paris Saclay (PS) Orsay Paris France; ^3^ Institut des Matériaux Poreux De Paris ESPCI CNRS ENS Université Paris Sciences & Lettres Paris France; ^4^ Department of Chemistry Korea Advanced Institute of Science and Technology (KAIST) Daejeon Republic of Korea; ^5^ AI Co‐Research & Education for Innovative Drug Institute (AI‐CRED Institute) Korea Advanced Institute of Science and Technology (KAIST) Daejeon Republic of Korea; ^6^ Gulf University For Science and Technology (GUST) Hawally Kuwait; ^7^ State Key Laboratory of Catalysis & Chinese Academy of Sciences Dalian Institute of Chemical Physics Dalian P. R. China; ^8^ Institute of Advanced Wear & Corrosion Resistant and Functional Materials College of Chemistry and Materials Science Jinan University Guangzhou P. R. China; ^9^ Research Institute of Frontier Science Southwest Jiaotong University Chengdu P. R. China; ^10^ College of Bioresources and Materials Engineering Shaanxi University of Science & Technology Xi'an P. R. China; ^11^ Energy and Environment Science & Technology Directorate Idaho National Laboratory Idaho Falls ID USA; ^12^ Department of Chemistry Colorado School of Mines Golden CO USA

**Keywords:** bridge‐site coordination, cysteine functionalization, electronic structure modulation, HER, PGM‐free electrocatalysts

## Abstract

Transition metal chalcogenides are among the most promising platinum group metal‐free (PGM‐free) catalysts for hydrogen production in a proton exchange membrane water electrolyzer (PEMWE). However, achieving both high activity and long‐term stability remains a critical challenge for their practical deployment. Here, we report a functionalization‐mediated synthesis of molybdenum sulfide, containing bridge‐coordinated Fe atoms (*b*‐Fe‐MoS_2_), achieving enhanced catalytic performance for the hydrogen evolution reaction (HER). Combined detailed spectroscopic and electrochemical characterizations revealed that cysteine functionalization promotes the anchoring of Fe atoms in a bridge‐like coordination environment on the MoS_2_ nanosheets, resulting in tunable electron density near the Fe sites and improved robustness of the atomic sites, as supported by our *operando* and post‐mortem investigations. The experimental results are complemented with density functional theory (DFT) calculations revealing that the unique coordination environment of Fe atoms modulates the hydrogen adsorption free energy. When integrated in a 25 cm^2^ catalyst‐coated membrane PEM water electrolyzer, *b*‐Fe‐MoS_2_ achieved remarkable performance, delivering 1.55 A cm^−2^ at 2 V and showed stable operation over 360 h following initial activation. These results demonstrate the effectiveness of the functionalization‐mediated approach for preparing robust HER catalysts and highlight the potential of bridge‐coordinated Fe–MoS_2_ cathodes for PGM‐free PEM water electrolyzers.

## Graphical abstract

We report a functionalization‐mediated strategy to synthesize robust iron (Fe) atoms selectively coordinated at bridge sites on molybdenum disulfide (MoS_2_) nanosheets. The resulting *b*‐Fe‐MoS_2_ catalyst exhibits enhanced activity and durability for the hydrogen evolution reaction in proton exchange membrane water electrolyzers compared to atop‐coordinated Fe sites (*a*‐Fe‐MoS_2_).

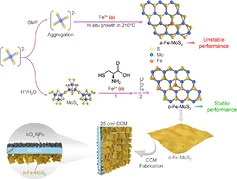

## 1 Introduction

Hydrogen (H_2_) is currently produced primarily through steam reforming of natural gas, a process reliant on finite fossil fuels and responsible for substantial carbon emissions [1, 2, 3]. In response to climate change and the urgent need for sustainable energy solutions, the European Union has set a target to produce 20 million tons of green hydrogen annually by 2030 [4]. This ambitious goal underscores the demand for efficient, cost‐effective, and stable electrolysis systems capable of generating high‐purity hydrogen, a promising pathway for cutting emissions in end‐use sectors where direct electrification remains challenging [5]. Proton exchange membrane water electrolysis (PEMWE) is an important technology for efficient and sustainable hydrogen production [6, 7], offering the potential to reduce emissions in transportation and seamless integration with intermittent renewable energy sources [8]. PEMWE presents several benefits, such as generating ultra‐pure hydrogen (99.999%) at pressures up to 30 bar, enabling a compact system design, and operating across a broad range of conditions [9]. The widespread adoption of PEMWE is critically hindered by its reliance on platinum group metals (PGM) such as iridium and platinum as electrocatalysts, despite PGM accounting for only about 8% of a PEMWE stack and 5% of the overall system cost; their limited availability could impede large‐scale implementation and hinder the wide commercialization of this technology [10, 11]. Advancing PEMWE technology and developing PGM‐free catalysts are crucial for achieving sustainable hydrogen production and reducing dependence on fossil fuels, thereby supporting global efforts to mitigate climate change and transition to a more sustainable energy future. However, the highly corrosive acidic environment of PEMWE, requiring materials that endure low pH, further restricts options to scarce and costly transition metals, highlighting the urgent need to develop efficient and durable PGM‐free alternatives to enhance system sustainability and reduce costs [12, 13, 14].

Transition metal chalcogenides (TMCs) represent a promising class of PGM‐free and low‐cost HER electrocatalyst [15, 16, 17, 18, 19]. TMCs can serve as a versatile platform for further modifications, such as heteroatom doping or single‐atom incorporation/coordination. In particular, the integration of single atomic active sites has been regarded as a promising strategy for decreasing the amount of transition metals and tuning the binding ability with hydrogen. The rational design and control of the coordination environment of the individual single atomic sites bring additional synthetic challenges, limiting practical developments. TMCs, in particular molybdenum sulfide (MoS_2_), have shown different stabilities depending on the structure [20], which further complicates the use of MoS_2_ as a support for single atoms. Although amorphous molybdenum sulfide has gained considerable interest due to its good HER catalytic activity, amorphous cluster‐like MoS_3‐x_ phases are prone to reconstruction and degradation during the HER, driven by sulfur depletion and the formation of undercoordinated Mo sites [21, 22, 23]. Molybdenum sulfide ‐based catalysts have rarely been demonstrated in large‐area electrolyzer cells (>25 cm^2^). Most reported systems are restricted to small active areas (≤5 cm^2^) and utilize catalyst‐coated substrate architectures (CCS, e.g., porous transport electrodes, PTEs, or gas diffusion electrodes, GDEs), which introduce considerable interfacial resistance and do not capitalize on the enhanced active surface area and improved interfacial integration achievable with catalyst‐coated membranes (CCM).

**FIGURE 1:**
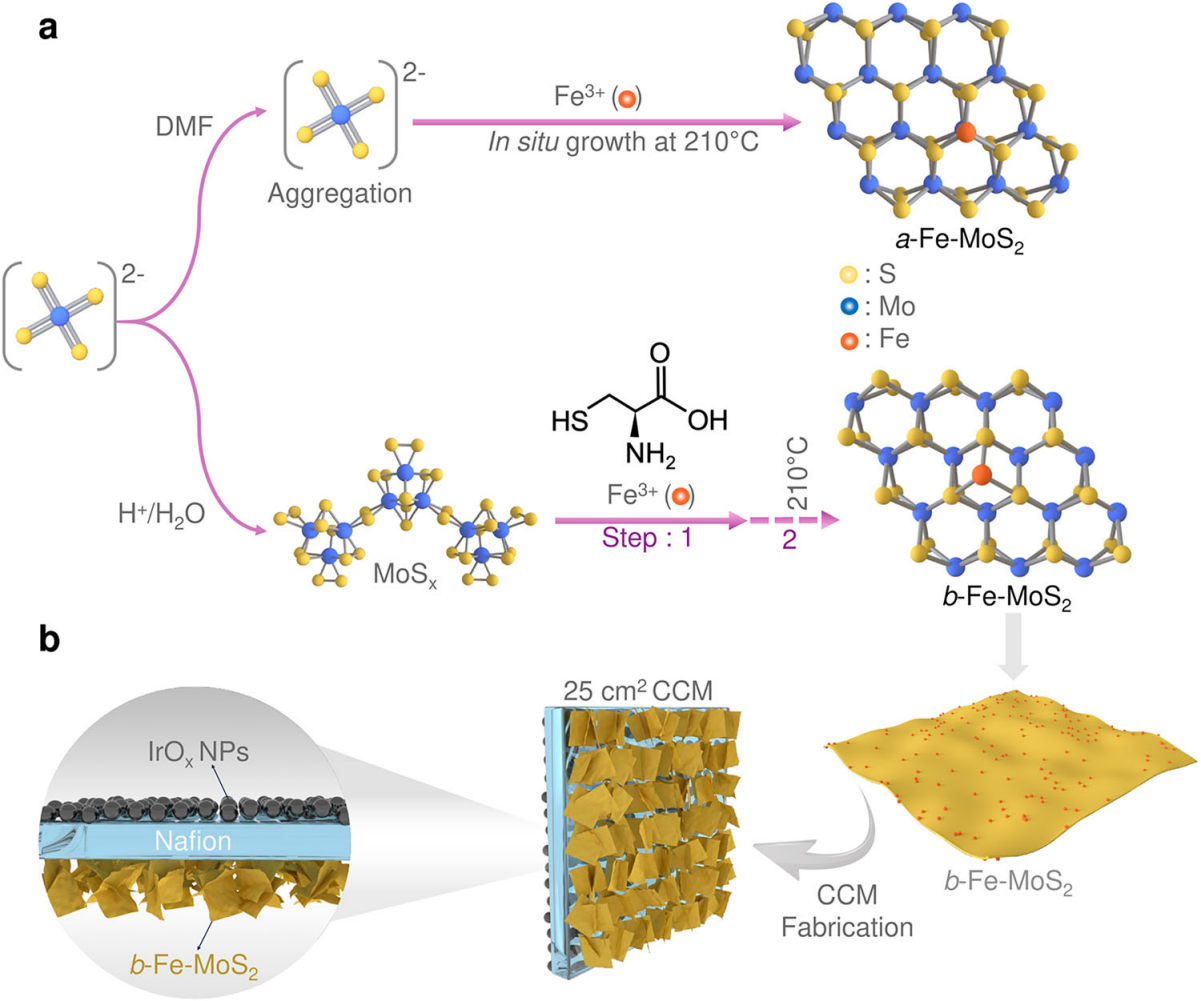
(a) Functionalization‐mediated synthesis strategy for generating bridge‐coordinated Fe atoms (*b*‐Fe‐MoS_2_) and atop‐coordinated Fe atoms (*a*‐Fe‐MoS_2_) on MoS_2_ nanosheets. (b) Schematic illustration of the CCM configuration comprising an IrO_x_ anode, a Nafion membrane, and a *b*‐Fe‐MoS_2_/carbon cathode layer containing Nafion ionomer.

To address these challenges, we report a bridge‐coordinated Fe atomic motif on MoS_2_ achieved through a functionalization‐mediated synthesis strategy. This approach ensures precise anchoring and stabilization of Fe atoms during the MoS_x_‐to‐MoS_2_ transformation, paving the way for scalable and high‐performance integration in practical devices (Figures 1a and S1a, Note 1). By taking advantage of the strong interactions of cysteine with defective molybdenum sulfide, we initially stabilize Fe atoms at the surface through coordination. Subsequent mild thermal annealing decomposes the cysteine ligand, leaving the Fe atoms anchored on the surface of the MoS_2_ nanosheets. During this process, the cysteine ligand releases sulfur, which not only stabilizes Fe atoms but also promotes their binding at bridge sites on MoS_2_ (*b*‐Fe‐MoS_2_). *Operando* characterization combined with theoretical investigations confirms the structural robustness of this motif and elucidates its optimized hydrogen adsorption behavior, which underpins enhanced HER performance. Density functional theory (DFT) reveals that the inclusion of bridge‐coordinated iron atoms on MoS_2_ enables tuning of the hydrogen binding energy. The free energy of hydrogen adsorption was estimated to be 0.21 eV for bridge‐coordinated Fe close to the optimal *atop* coordination. Importantly, stability testing indicates markedly improved stability of the bridge‐coordinated Fe compared to *atop* coordinated Fe atoms. Beyond fundamental insights, device‐level evaluations further validate the practical relevance and applicability of this atomic‐scale catalyst design under realistic operating conditions. To demonstrate the potential of our synthetic route, we successfully fabricated a 25 cm^2^ CCM (Figure 1b) and integrated it into a PEM water electrolyzer (PEMWE). The resulting *b*‐Fe‐MoS_2_ cathode delivers 1.55 A cm^−2^ at 2 V. Importantly, the system maintains improved stability under dynamic‐operation stress test, underscoring the potential of this functionalization‐mediated route for scalable, high‐performance MoS_2_‐based electrolyzers.

## 2 Results and Discussion

### 2.1 Structural Investigation of Prepared *b‐*Fe‐MoS_2_

Molybdenum sulfide with high sulfur content (MoS_x_, x > 2) was synthesized by the acidification of ammonium tetrathiomolybdate using ascorbic acid. Scanning electron microscopy (SEM) images and energy‐dispersive x‐ray spectroscopy (EDS) analyses suggested a Mo:S ratio of 1:3.7, larger than the expected stoichiometry of MoS_3_ previously reported [24, 25, 26]. X‐ray diffraction (XRD) shows a broad peak at ∼14° [27], indicating an amorphous material likely based on [Mo_3_S_13_]^2−^ units (referred to as MoS_x_) [28]. After functionalization with the Fe^3+^ and cysteine (referred to as MoS_x__Fe_Cyt), the new matrix remains amorphous, retaining the weak (002) peak at ∼14° [29, 30], while a new peak at ∼18° is visible and attributed to the presence of cysteine (Note 1, Figures S1–S3). *b*‐Fe‐MoS_2_ was then obtained by solvothermal treatment of MoS_x__Fe_Cyt at 210°C. We also synthesized Fe‐MoS_2_ samples in the absence of cysteine to induce the formation of atop‐coordinated Fe atoms on the MoS_2_ basal plane: *a*‐Fe‐MoS_2_ [31]. XRD shows an intense (002) peak corresponding to an interlayer spacing of 0.93 nm, which is shifted relative to that of 1T′‐MoS_2_ nanosheets (Figure S3b). This finding indicates that the observed increase in the interlayer space may be attributed to Fe incorporation into MoS_2_ nanosheets [31]. In addition, Raman and x‐ray photoelectron spectroscopy (XPS) (Figures S3c,d) reveal the presence of 1T’ and 2H phases, confirming a mixed phase—consistent with low‐temperature synthesis (Note 2).

**FIGURE 2:**
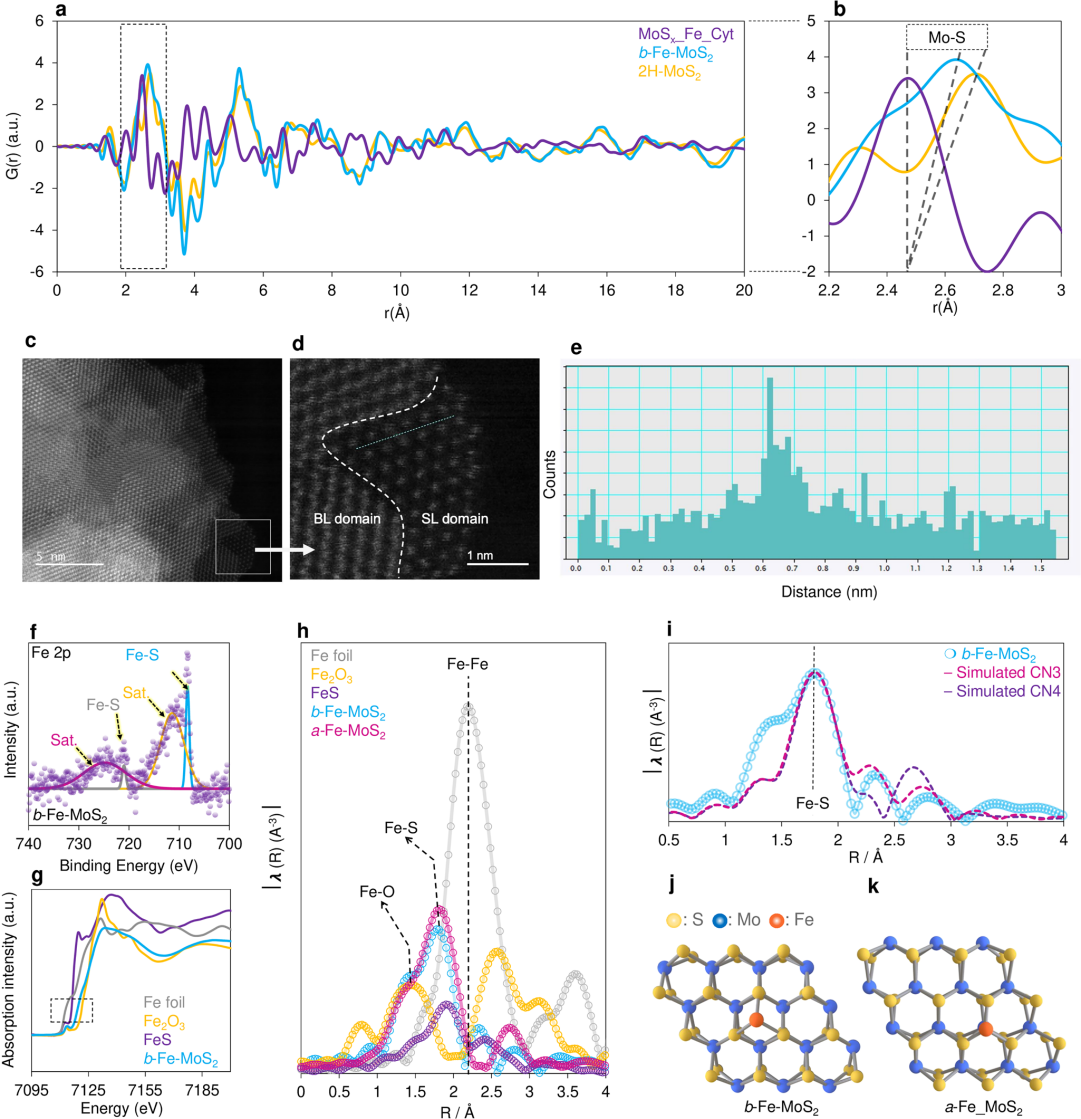
(a and b) Pair distribution function (PDF) profiles derived from HE‐XRD measurements of MoS_x__Fe_Cyt, *b*‐Fe‐MoS_2_, and pristine 2H‐MoS_2_, together with an enlargement of the short‐range correlation region. (c) Atomic‐resolution STEM image of *b*‐Fe‐MoS_2_ nanosheet showing locally ordered lattice domains. (d) Enlarged STEM image of the selected region in panel **c**, highlighting the boundary between bilayer (BL) and single‐layer (SL) domains; the line indicates the position used for intensity‐profile analysis. (e) Line intensity profile scans extracted along the blue line indicated in panel **d**, illustrating variations in local atomic contrast within the thin nanosheet region. (f) High‐resolution Fe 2p XPS spectrum of *b*‐Fe‐MoS_2_. (g) Fe K‐edge XANES spectra of *b*‐Fe‐MoS_2_ and reference compounds. (h) Fourier‐transformed EXAFS spectra at the Fe K edge. (i) Comparison of the experimental FT‐EXAFS spectrum of *b*‐Fe‐MoS_2_ with simulated spectra based on CN3 bridge‐coordinated and CN4 atop‐coordinated Fe configurations. (j and k) DFT‐optimized structural models corresponding to the *b*‐Fe‐MoS_2_ (CN3) and *a*‐Fe‐MoS_2_ (CN4) configurations.

To probe the local atomic structure of *b*‐Fe‐MoS_2_, we carried out high‐energy x‐ray diffraction (HE‐XRD, Figure 2a,b). The pair distribution function (PDF) reveals short‐ and medium‐range atomic structures in disordered materials beyond Bragg diffraction [32]. The MoS_x__Fe_Cyt structure shows typical features of a material that lacks long‐range order while maintaining well‐defined short‐range correlations compared to *b*‐Fe‐MoS_2_ and crystalline 2H‐MoS_2_ (Figure 2a). The maximum atomic correlation length in MoS_x__Fe_Cyt is around 12 Å, indicative of randomly connected, intrinsically disordered clusters rather than an extended periodic network [32]. The PDF analysis of MoS_x__Fe_Cyt exhibits a prominent Mo^…^S peak at 2.48 Å, consistent with the structure reported in prior studies, and a Mo^…^Mo correlation at approximately 2.93 Å, slightly longer than that observed in (NH_4_)_2_Mo_3_S_13_ [32]. This is also in line with previous Fourier‐transform extended x‐ray absorption fine structure (EXAFS) spectra of MoS_2_‐based materials, with characteristic peaks at ∼2 and ∼3 Å corresponding to Mo^…^S and Mo^…^Mo bonds, respectively [33].

Comparative PDF analyses of MoS_x__Fe_Cyt with *b*‐Fe‐MoS_2_ and crystalline 2H‐MoS_2_ reveal longer Mo^…^S bond lengths of 2.62 and 2.72 Å, respectively, and an extended periodicity up to 20 Å (Figure 2a). These observations suggest crystallization of the *b*‐Fe‐MoS_2_ framework, likely arising from structural reorganization during Fe incorporation and the transformation of MoS_x__Fe_Cyt. The presence of a peak at 2.62 Å in *b*‐Fe‐MoS_2_ suggests the formation of analogous Fe/Mo–S species, further supporting the incorporation of Fe into the MoS_2_ lattice. This interpretation is supported by prior PDF studies of CoMo_6_S_24_, which exhibit peaks at 2.42, 2.75, and 3.36 Å, assigned to nearest‐neighbor Co/Mo^…^S, Mo^…^Mo, and Mo^…^Mo interactions, respectively [34]. Collectively, these results demonstrate the sensitivity of PDF analysis in probing local atomic arrangements and emphasize its role in clarifying the complex structure of *b*‐Fe‐MoS_2_.

We used a series of transmission electron microscopy techniques (TEM), including high resolution transmission electron microscopy (HRTEM) and high‐angle annular dark field (HAADF) scanning transmission electron microscope (STEM) to visualize the atomic structure of *b*‐Fe‐MoS_2_ (Figure S4 and Note 3). Our observations reveal distinct layered domains with an interlayer spacing of 0.94 nm, consistent with lattice modification caused by Fe doping and close to that observed by XRD (Figure S4a,b). Atomic‐resolution STEM–EDS imaging of multilayer *b*‐Fe‐MoS_2_ nanosheets indicates ordered lattice domains and confirms the presence of Fe within the analysed regions (Figures S4c–g). The *b*‐Fe‐MoS_2_ sample consists in multilayer‐stacked nanosheets and the fact that Mo and Fe atoms share similar Z number makes the direct observation of individual atoms challenging. To reduce projection, overlap from stacked nanosheets and examine the local Fe distribution more clearly, a thin monolayer‐like region was subsequently analyzed (Figure 2c,d). The Fe‐related contrast observed in the monolayer region is consistent with dispersed coordinated Fe sites incorporated within the MoS_2_ framework. The intensity line profiles extracted from the HAADF‐STEM image confirm the presence of isolated atoms within the MoS_2_ lattice and provide complementary evidence for local Fe incorporation (Figure 2e). High‐resolution x‐ray photoelectron spectroscopy (XPS) analysis of the Fe 2p region further supports the existence of Fe─S bonds in *b*‐Fe‐MoS_2_, with Fe‐related components observed at 708.1 eV for Fe 2p_3/2_ and 720.7 eV for Fe 2p_1/2_, together with satellite contributions at 710.05 and 723.15 eV. No detectable metallic Fe contribution—expected near ∼706.7–707.0 eV—is observed (Figure 2f). Detailed assignments and discussions of these spectral features are provided in Note 3.

To elucidate the electronic structure and local coordination environment of Fe atoms, we performed x‐ray absorption near‐edge spectroscopy (XANES) and extended x‐ray absorption fine structure (EXAFS) measurements on *b*‐Fe‐MoS_2_ at the Fe‐K edge (Figure 2g). The XANES spectrum of *b*‐Fe‐MoS_2_ revealed an absorption‐edge energy position higher than that of metallic Fe and intermediate between Fe_2_O_3_ and FeS, indicating an oxidation state of Fe between +2 and +3. The average oxidation state of Fe in *b*‐Fe‐MoS_2_ is estimated to be approximately +2.18 by interpolation between the FeS and Fe_2_O_3_ reference compounds (Figure S5). Fourier‐transformed EXAFS (FT‐EXAFS) shows a main peak for *b*‐Fe‐MoS_2_ at ∼1.79 Å (without phase correction), consistent with the Fe─S coordination found in Fe─S systems (Figure 2h). Crucially, the absence of a Fe–Fe scattering feature at ∼2.20 Å rules out the presence of Fe clusters, supporting atomically dispersed Fe sites on MoS_2_. Quantitative fitting of the Fe K‐edge EXAFS reveals an average coordination number of ∼2.60 ± 0.22 for Fe─S and 1.32 ± 0.31 for Fe─O (Table S1). This value suggests that the Fe atoms in *b*‐Fe‐MoS_2_ are coordinated by three lattice S atoms corresponding to a coordination in a bridge position on the MoS_2_ nanosheets. Additional Fe─O signals are attributed to the presence of oxygen atoms from absorbed hydroxyl or water [35] or from the presence of axial oxygen atoms bonded to Fe likely due to air oxidation [31]. Importantly, this bridge‐like coordination differs from Fe atoms occupying *atop* sites on MoS_2_ nanosheets, as well as from similar reported catalysts. The simulated EXAFS spectra show that the experimental data are in better agreement with the CN3 model than with the CN4 model corresponding to *bridge* and *atop* configuration, respectively (Figure 2i, Table S1). The simulated EXAFS spectrum of the bridge‐like Fe configuration reproduces the main features of the experimental Fe K‐edge EXAFS spectrum of *b*‐Fe‐MoS_2_. In contrast, the atop coordination model (*a*‐Fe‐MoS_2_), fails to capture the characteristic spectral features of *b*‐Fe‐MoS_2_, thereby providing evidence for the presence of a bridge‐like Fe coordination environment in *b*‐Fe‐MoS_2_. The corresponding theoretical EXAFS path simulations were performed based on the DFT‐optimized CN3 and CN4 structural models (Figure 2j,k).

### 2.2 Electrochemical Performance of Prepared Electrodes‐Based *b*‐Fe‐MoS_2_

**FIGURE 3:**
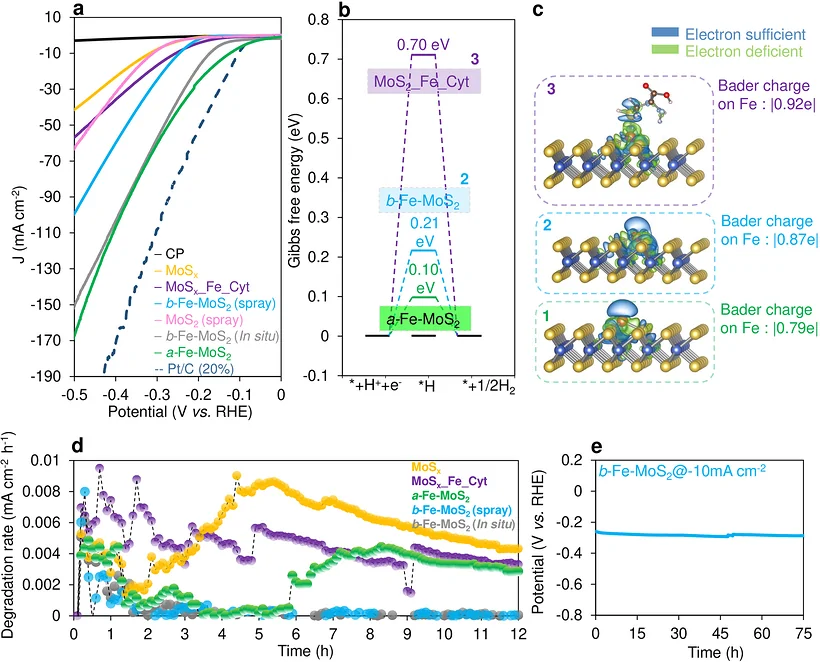
(a) HER polarization curves of MoS_2_‐based catalysts measured in 0.5 M H_2_SO_4_. (b) Calculated Gibbs free energies of hydrogen adsorption for Fe‐coordinated MoS_2_ catalyst models. (c) Charge‐density‐difference maps and calculated Bader charges illustrating charge redistribution around the Fe sites. (d) Degradation rates of the investigated catalysts under HER conditions. (e) Chronopotentiometric stability of *b*‐Fe‐MoS_2_ at −10 mA cm^−2^. The *a*‐Fe‐MoS_2_ structural motif was previously reported in Ref. [31]; its HER performance was evaluated in this work under identical experimental conditions as those used for *b*‐Fe‐MoS_2_, to allow for a direct comparison between iron‐based catalysts.

The electrochemical properties of *b*‐Fe‐MoS_2_ were first evaluated in a 0.5 M H_2_SO_4_ electrolyte by loading the catalysts on a carbon paper (CP) support (Figure S6). The linear sweep voltammetry curves (LSV) reveal that the *b*‐Fe‐MoS_2_ catalyst exhibits the lowest onset potential compared to MoS_x__Fe_Cyt and MoS_x_. MoS_x_ _Fe_Cyt and *b*‐Fe‐MoS_2_ catalysts display an overpotential between 250–170 mV versus RHE at a current density of ‐10 mA cm^−2^, outperforming MoS_x_, which shows an overpotential of 320 mV versus RHE (Figure 3a). At an overpotential of 500 mV, the responses of MoS_x_ and MoS_x__Fe_Cyt become comparable, whereas *b*‐Fe‐MoS_2_ maintains superior catalytic performance. A control MoS_2_ sample subjected to the same solvothermal treatment without Fe was synthesized and exhibited lower performance, confirming that the enhanced activity primarily arises from Fe incorporation. The activity and the stability of *b*‐Fe‐MoS_2_ and MoS_x__Fe_Cyt were also examined using a glassy carbon support (Note 4, Figures S7a–f). These experiments confirmed that the presence of bridged‐coordinated Fe significantly enhances HER activity. To optimize the physical and electrical coupling between the catalyst and the carbon support, we also synthesized *b*‐Fe‐MoS_2_ directly on the CP during the solvothermal synthesis (*b*‐Fe‐MoS_2_‐CP). This in situ growth approach led to marked improvements in catalytic performance, particularly in terms of current density (Figure 3a). The superior performance is attributed to the superior coupling with carbon support, which improves electrical conductivity and promotes better accessibility of active sites. This improvement is further reflected in the reduced Tafel slope, which decreases from 118 to 103 mV dec^−1^ for spray coated and in situ grown samples, respectively (Figure S7g). In addition, electrochemical impedance spectroscopy (EIS) measurements show a lower charge‐transfer resistance (R_ct_) for the in situ‐grown electrode (*b*‐Fe‐MoS_2_‐CP) than for the spray‐coated *b*‐Fe‐MoS_2_, consistent with improved catalyst–substrate electrical contact and more efficient interfacial charge transfer (Figure S7h). We examine the morphology of the in situ‐grown *b*‐Fe‐MoS_2_ on CP substrate using SEM‐EDS (Figures S7i–j), and we observed uniform growth of nanosheets on the carbon paper fibers, while EDS mapping confirmed the homogeneous distribution of *b*‐Fe‐MoS_2_. In addition, Figure S7k shows a TEM image of ultrathin layered nanosheets detached from the GDL with an expanded interlayer spacing of approximately 0.93 nm, comparable to that measured for the powder catalyst (0.94 nm) and consistent with the XRD‐derived interlayer spacing (0.93 nm).

To elucidate the origin of the performance enhancement, we examined the role of the Fe coordination atomic sites. A comparison with *a*‐Fe‐MoS_2_ system, where Fe atoms are coordinated in *atop* positions on MoS_2_ nanosheets, revealed subtle but noticeable differences in current density. These differences are attributed to variations in Fe coordination geometry, which directly influences catalytic activity. EXAFS‐derived structural models were employed as inputs for density functional theory (DFT) calculations to determine the hydrogen adsorption free energy ($\Delta G_{H^{*}}^{o}$). *b*‐Fe‐MoS_2_ exhibited a value of 0.21 eV, while *a*‐Fe‐MoS_2_ displayed a lower value of 0.10 eV, consistent with their respective HER activities (Figure 3a,b, Note 5). The Mo 3d XPS spectrum of *b*‐Fe‐MoS_2_ displays broadened features and shifted components relative to the 2H‐MoS_2_ reference. This is consistent with the formation of electronically modified Mo environments and increased electron density around Mo sites (Note 2 and Figure S3d). The Fe 2p binding energy in *b*‐Fe‐MoS_2_ (Figure 2f) indicates a higher Fe oxidation state, consistent with the Fe K‐edge XANES data (Figure 2g). Based on our XANES measurements, we estimated the average Fe oxidation state in *b*‐Fe‐MoS_2_ to be +2.18 close to that of *a*‐Fe‐MoS_2_ (+2.25) and different from +2 or +3. We also calculated the Bader charge of MoS_x__Fe_Cyt and compared it to (a‐) or *b*‐Fe‐MoS_2_ (Figure 3c). The Bader charge analysis revealed a charge transfer of |0.87e| for *b*‐Fe‐MoS_2_, which is slightly lower than that of MoS_2__Fe_Cyt (|0.92e|), but higher than that of *a*‐Fe‐MoS_2_ (|0.79e|). While direct correlation between Bader charge and oxidation state is usually difficult to draw, these differences reflect distinct clear electronic environments for Fe in each system, which directly impact their electrocatalytic performance and stability in HER (Figure 3a,b). Collectively, our data confirm the higher oxidation degree of Fe atoms in the bridge and atop position (a detailed description is provided in Note 5).

### 2.3 Structural Stability of *b*‐Fe‐MoS_2_

**FIGURE 4:**
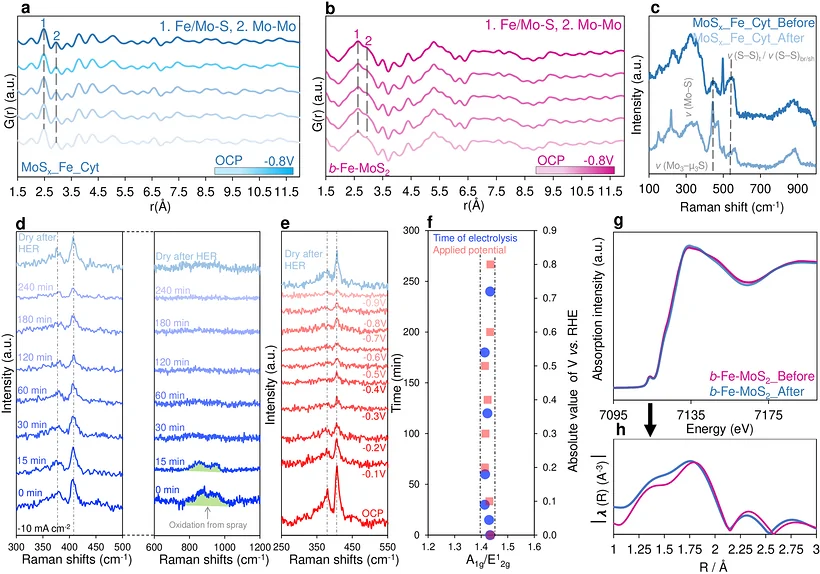
(a and b) *Operando* PDF profiles derived from HE‐XRD measurements of MoS_x__Fe_Cyt and *b*‐Fe‐MoS_2_ recorded at different applied potentials during HER. (c) Raman spectra of MoS_x__Fe_Cyt before and after HER. (d) Time‐dependent in situ Raman evolution of *b*‐Fe‐MoS_2_ during HER at −10 mA cm^−2^. (e) Potential‐dependent in situ Raman spectra of *b*‐Fe‐MoS_2_. (f) Evolution of the Raman A_1g_/E^1^_2g_ intensity ratio as a function of electrolysis time and applied potential. (g) Fe K‐edge XANES spectra and (h) FT‐EXAFS spectra of *b*‐Fe‐MoS_2_ before and after HER.

To assess the long‐term stability, we evaluated the HER performance under continuous applied potential over 12 h (Note 5). With a small degradation rate (Figure 3d), *b*‐Fe‐MoS_2_ stands out as the most stable catalyst over 70 h (Figure 3e). The degradation rate of *b*‐Fe‐MoS_2_ was more than two orders of magnitude lower than that of *a*‐Fe‐MoS_2_, underscoring the outstanding durability conferred by the bridge‐coordinated Fe sites. These results highlight the critical role of Fe site configuration in determining both activity and stability: while Fe atoms at *atop* sites demonstrate higher initial activity, they suffer from reduced stability compared to the *bridge*‐coordinated Fe sites (Note 5). To rationalize these observations, we employed HE‐XRD coupled with pair PDF analyses. At open‐circuit potential (OCP), the PDF analysis revealed a periodic structure extending up to 20 Å in *b*‐Fe‐MoS_2_ (Figure S8a,b). *Operando* HE‐XRD patterns were collected under decreasing electrode bias (Figure 4a,b). The results confirmed that all peaks for *b*‐Fe‐MoS_2_ and MoS_x__Fe_Cyt remained at identical positions, suggesting structural stability of the catalysts under HER conditions.

To further probe potential structural reconstructions, we monitored the evolution of Raman spectra for MoS_x__Fe_Cyt (Figure 4c). The decrease in signals corresponding to bridging and terminal disulfide bonds suggests a loss of terminal ligands and potential alterations in the [Mo_3_] cluster geometry. These results point out that cysteine functionalization alone does not improve MoS_x_ stability. However, the good stability observed by HE‐XRD may be due to the presence of the carbon support in the experimental set‐up, which may also contribute to the stabilization of the active sites. In contrast, in situ Raman of *b*‐Fe‐MoS_2_ confirmed the preservation of the MoS_2_ structure (Figure 4d–f). In situ Raman measurements under chronopotentiometry (−10 mA cm^−2^, 5 h) reveal an initial surface oxidation that rapidly disappears with time (Figures 4d and S8c). Raman measurements at different applied potentials further confirm the structural stability of the catalyst under operating conditions (Figure 4e). These results (Figure 4f) with extended chronopotentiometry (−10 mA cm^−2^, 100 h) coupled with ex situ XPS, show that the MoS_2_ framework remains preserved after electrolysis (Figure S9a). Contributions from Mo^6+^ are detected in XPS and attributed to air exposure of the samples before XPS analyses, while the Mo^4+^ signals are assigned to MoS_2_ (Figure S9a). This is also supported by the fact that the oxidized signal for the samples remains virtually constant from t = 0 h to t = 100 h. To finally conclude on the evolution of the coordinated Fe atoms, we performed post‐mortem XANES and EXAFS on *b*‐Fe‐MoS_2_. Our results show that both spectra are nearly identical to that of pristine samples with similar first‐shell coordination and a stable Fe oxidation state. Taken together, these results highlight the robustness of the bridge coordination Fe sites during catalytic operation (Figure 4g,h). HRTEM‐STEM/EDS mapping before and after HER further confirms that the MoS_2_ framework and the Fe dispersion are largely preserved under electrocatalytic conditions (Figures S9b–d and Note 6).

### 2.4 Evaluating the Electrochemical Performance in a PEMWE Cell

**FIGURE 5:**
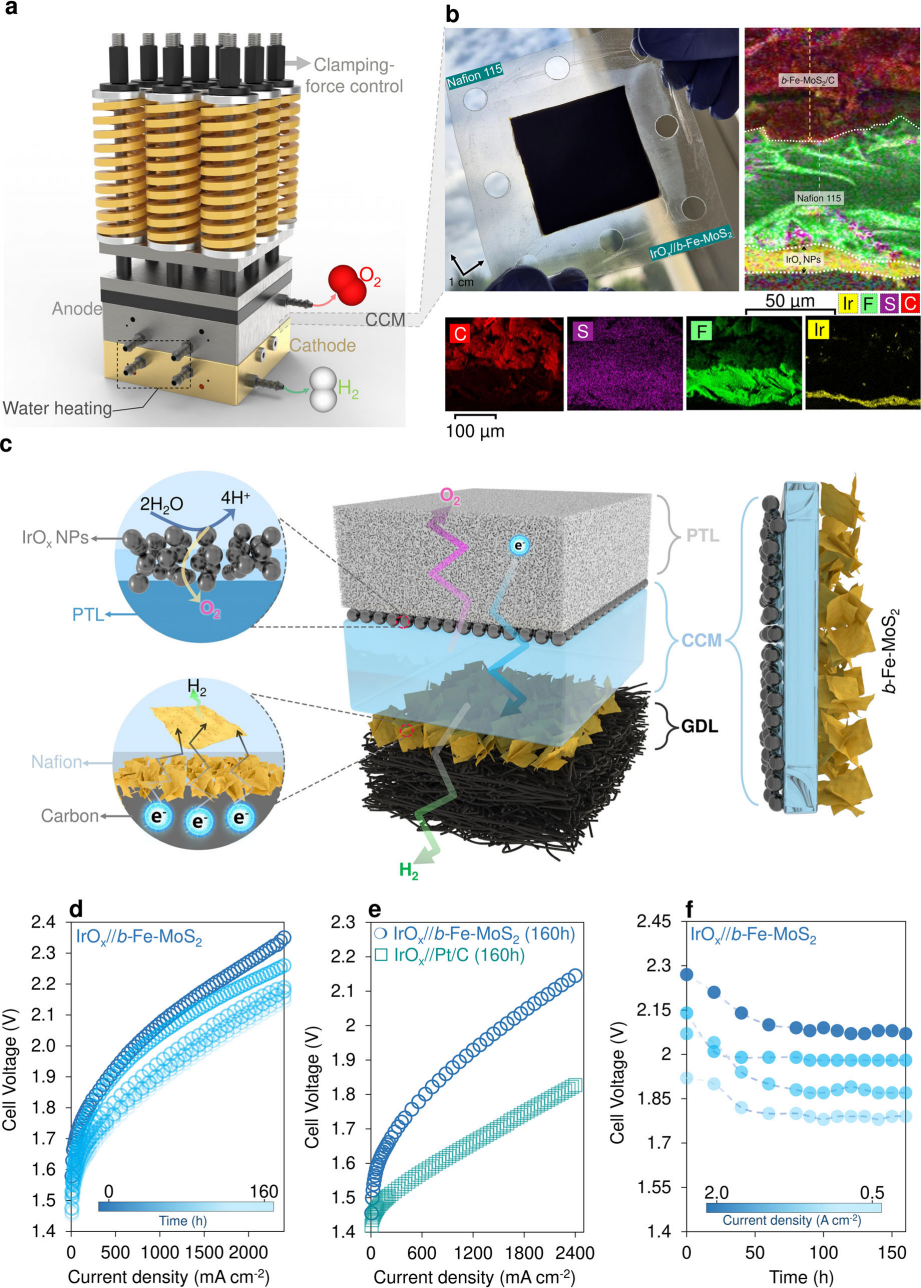
(a) Schematic representation of the PEM water electrolyzer cell. (b) Photograph of the prepared CCM and cross‐sectional SEM–EDS elemental mapping of the membrane–electrode assembly. (c) Schematic representation of the CCM‐based PEMWE architecture, showing the IrO_x_ anode, Nafion membrane, and *b*‐Fe‐MoS_2_ cathode layer. (d) Polarization curves of the IrO_x_//*b*‐Fe‐MoS_2_ PEMWE recorded at different times during the dynamic‐operation stress test. (e) Comparison of the polarization curves of IrO_x_//*b*‐Fe‐MoS_2_ and IrO_x_//Pt/C cells after 160 h of operation. (f) Evolution of cell voltage at the indicated current densities, extracted periodically from polarization curves at selected diagnostic current densities during the 160‐h dynamic‐operation stress test.

**FIGURE 6:**
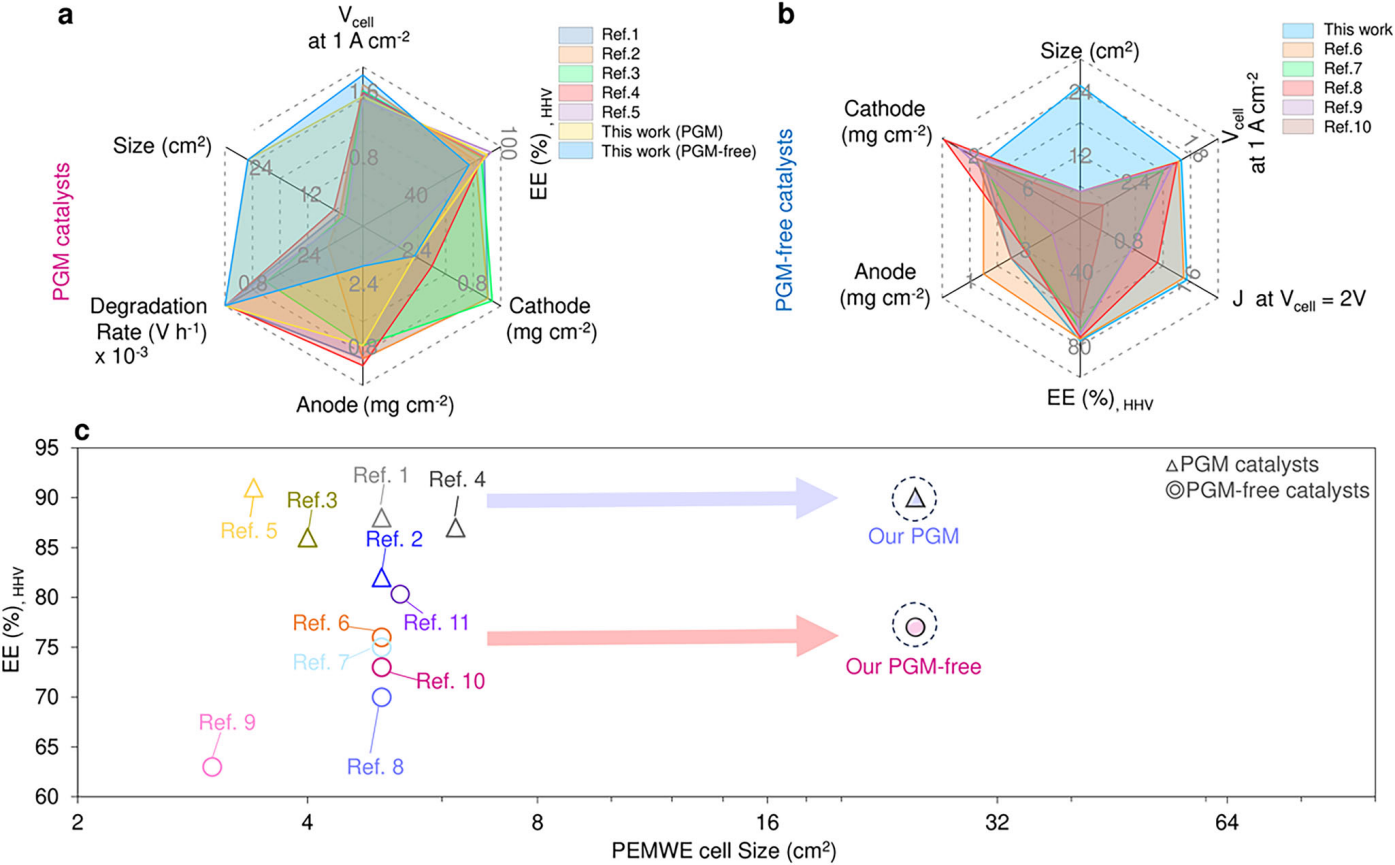
Benchmarking of PEM water electrolyzers employing PGM or PGM‐free cathode catalysts. (a and b) Radar plots comparing the present IrO_x_//b‐Fe‐MoS_2_ system with previously reported PEMWE configurations in terms of active area, catalyst loading, energy efficiency, current density, voltage requirement, and degradation rate, where reported. (c) Comparison of energy efficiency and active cell area for PEMWEs employing PGM or PGM‐free cathodes. Data and literature references are provided in Table S2.

We assessed the properties of *b*‐Fe‐MoS_2_ catalyst in a PEMWE cell (Figure 5a, Note 7) [36]. CCM was prepared by decal transfer onto Nafion N115 (Figure 5b,c) and assembled with an IrO_x_ anode and either *b*‐Fe‐MoS_2_ or Pt/C cathodes. Catalyst loadings were fixed at 2.5 mg cm^−2^ for IrO_x_ and *b*‐Fe‐MoS_2_/Carbon (50 wt%), or 0.5 mg cm^−2^ for Pt/C (60 wt%). The Pt/C reference showed good performance, confirming the robustness of the CCM fabrication and the reliability of our single‐cell PEMWE setup. Dynamic‐operation stress tests over 160 h were performed, combining several stress factors to simulate current intermittency and dynamic operation at 80°C (A detailed description is provided in Note 7). The cell was operated in a closed‐loop configuration with batch water recirculation, enabling up to 24 h of continuous operation. Under these conditions, the IrO_x_//Pt/C cell remained stable, with unchanged polarization curves and constant HFR (∼105 mΩ cm^2^) (Figures S10 and S11). Applying the same stress test protocol to *b*‐Fe‐MoS_2_ (Figure 5d) revealed an initial voltage drop followed by continuous improvement and stabilization (Figures S12 and S13). The initial voltage drop is attributed to mass‐transport limitations from the thicker cathode catalyst layer, consistent with signals from the low frequency region from our EIS trends at current densities >200 mA cm^−2^ (Figures S12 and S13b,c). After ∼20 h, effects of limited diffusion diminish and *b*‐Fe‐MoS_2_ reaches stable operation and remained stable for 160 h with essentially unchanged HFR‐free polarization curves (Figures 5e,f and S14). Therefore, these observations suggest that the initial cell behavior is not associated with catalyst structural changes but is consistent with conditioning of the catalyst layer and its interface with the Nafion membrane. In addition, a test of 360 h at 400 mA cm^−2^ was performed (before starting, the CCM was activated at 0.5 A cm^−2^ for 32 h) and indicates promising stability of the PEMWE with a retention of the potential of 96% (Figure S15). The catalyst achieved 1.55 A cm^−2^ at 2.0 V (Figure 5e,f), ranking among the best stable MoS_2_‐based PEMWE cathodes (Figure 6, Note 7).

Figure 6 and Table S2 illustrate a comparative evaluation of various PEMWEs using both PGM and PGM‐free catalysts (cathode). We used radar plots to benchmark key performance metrics. Figure 6a further confirms the promising properties of *b*‐Fe‐MoS_2_ when comparing it against other PGM catalysts (PEM1–PEM5), also highlighting improved stability and reduced degradation rate (160 h dynamic‐operation stress test was used to calculate the degradation rate). In Figure 6b, among the PGM‐free cathode systems (PEM6–PEM10), our work demonstrates enhanced performance, achieving the highest energy efficiency with the largest active cell area (25 cm^2^), indicating excellent material utilization and scale‐up potential. We note that while most PGM‐free‐based systems achieve efficiency of ∼65%–80% (relative to the H_2_ higher heating value, HHV), they are limited to small cell sizes and CCS architectures (Figure 6c). In contrast, our PGM‐free design (cathode side) effectively addresses an important scale‐up challenge by demonstrating a 25 cm^2^ CCM cell, while maintaining high efficiency (∼77%). Overall, considering both performance and active area, the resulting cell ranks among the best PGM‐free PEMWE systems (cathode side) reported to date.

## 3 Conclusions

In this work, we develop a functionalization‐mediated strategy that enables the formation of a well‐defined bridge‐coordinated Fe atomic configuration within the MoS_2_ lattice via molecular functionalization. This approach promotes the incorporation of Fe atoms at bridge sites, resulting in enhanced catalytic activity and improved long‐term stability. Furthermore, we identify and elucidate this atomic motif, establishing the structure–property–stability relationship that governs its behavior under HER conditions. Electrochemical measurements under acidic conditions demonstrated that *b*‐Fe‐MoS_2_ exhibits good stability, with a degradation rate two orders of magnitude lower than that of *a*‐Fe‐MoS_2_ and reduced onset potentials. These performance gains are attributed to the modulation of hydrogen adsorption energy induced by Fe in a bridge position on MoS_2_, as supported by DFT calculations. Comprehensive *post‐mortem* and *operando* characterization, including EXAFS, XANES, in situ Raman and HE‐XRD, confirmed the structural robustness of the catalyst. Beyond the intrinsic HER properties of the bridge‐coordinated Fe sites, this work demonstrates the integration of *b*‐Fe‐MoS_2_ into a 25 cm^2^ catalyst‐coated membrane PEM water electrolyzer. Scaling from laboratory electrodes to a CCM device required management of the thicker non‐PGM cathode layer through the incorporation of conductive carbon, Nafion ionomer, and direct membrane integration by decal transfer. The resulting CCM system delivered performance comparable to that of conventional Pt/C systems, achieving a current density of 1.55 A cm^−2^ at 2 V and an impressive energy efficiency among the highest reported for non‐noble metal catalysts in PEM electrolyzers. Notably, the catalyst demonstrated more than 160 h of stability under dynamic‐operation stress test. This work highlights the potential of molecular functionalization to unlock novel coordination environments of Fe in MoS_2_ as a promising and scalable alternative to noble metals for green hydrogen production. Our findings open new directions for the rational design of catalysts and MoS_2_‐based CCM PEM water electrolyzers, identify *b*‐Fe‐MoS_2_ as a promising PGM‐free cathode platform, and highlight catalyst‐layer conductivity and membrane‐interface optimization as important directions for further PEMWE scale‐up.

## Author Contributions

**Zakaria Anfar**: conceptualization, methodology, writing – original draft, investigation. **Haitham Maslouh**: investigation. **Yuwei Yang**: investigation. **Mathilde Moderne**: investigation. **Jiefeng Liu**: investigation. **Loïc Assaud**: investigation, validation. **Mikhael Bechelany**: investigation, validation. **Chrystelle Salameh**: investigation, validation. **Kun Qi**: investigation, validation. **Yuying Meng**: investigation, validation. **Huali Wu**: investigation, validation. **Ji Li**: investigation, validation, methodology. **Nicholas M. Bedford**: investigation, validation, methodology. **Damien Voiry**: investigation, conceptualization, validation, methodology, writing – review and editing.

## Conflicts of Interest

The authors declare no conflicts of interest.

## Supporting information




**Supporting File**: The authors have cited additional references within the Supporting Information., Supplementary Notes 1–7, Figures 1–15, Tables 1–2 and References.

## Data Availability

The data that support the findings of this study are available from the corresponding author upon reasonable request.
