# Supplementary material for: Functionalization‐Mediated Synthesis of Bridge‐Coordinated Fe Atoms on MoS2 Nanosheets for PEM Water Electrolyzers
Source: Angew Chem Int Ed Engl. 2026 Jul 27;65(39):e6960943. doi: 10.1002/anie.6960943 (PMC13592318; doi:10.1002/anie.6960943)
Supplement: Supplementary file 1 [file ANIE-65-e6960943-s001.docx]

**Supporting information**

**Functionalization-Mediated Synthesis of Bridge-Coordinated Fe Atoms on MoS_2_ Nanosheets for PEM Water Electrolyzers**

Zakaria Anfar^[a]^*, Haitham Maslouh^[a], [b], [c]^, Yuwei Yang^[d], [e]^, Mathilde Moderne^[a]^, Jiefeng Liu^[a]^, Loïc Assaud^[c]^, Mikhael Bechelany^[a], [f]^, Chrystelle Salameh^[a]^, Kun Qi^[g]^, Yuying Meng^[h]^, Huali Wu^[i]^, Ji Li^[j]^, Nicholas M. Bedford^[k], [l]^, Damien Voiry^[a]^*

[a] Institut Européen des Membranes (IEM) – University of Montpellier (UM), CNRS, ENSCM, Montpellier. 34000 Montpellier France. *Email : [zakaria.anfar@umontpellier.fr](mailto:zakaria.anfar@umontpellier.fr) (Z. Anfar); damien.voiry@umontpellier.fr (D. Voiry)

[b] Institut de Chimie Moléculaire et des Matériaux d’Orsay (ICMMO) – Université Paris Saclay (PS), Paris. Orsay 91400 France.

[c] Institut des Matériaux Poreux de Paris, Université Paris Sciences & Lettres, ESPCI, CNRS, ENS, 10 rue Vauquelin, 75005 Paris, France

[d] Department of Chemistry, Korea Advanced Institute of Science and Technology (KAIST), Daejeon 34141, Republic of Korea

[e] AI Co-Research & Education for Innovative Drug Institute (AI-CRED Institute), Korea Advanced Institute of Science and Technology (KAIST), Daejeon 34141, Republic of Korea

[f] Gulf University for Science and Technology (GUST), Hawally 32093, Kuwait.

[g] State Key Laboratory of Catalysis & Chinese Academy of Sciences, Dalian Institute of Chemical Physics, Dalian, 116023 China

[h] Institute of Advanced Wear & Corrosion Resistant and Functional Materials, College of Chemistry and Materials Science, Jinan University, Guangzhou, 510632, China

[i] Research Institute of Frontier Science, Southwest Jiaotong University, Chengdu, 610031 PR China

[j] College of Bioresources and Materials Engineering, Shaanxi University of Science & Technology, Xi'an 710021, PR China

[k] Energy and Environment Science & Technology Directorate, Idaho National Laboratory, Idaho Falls, ID, 83415

[l] Department of Chemistry, Colorado School of Mines, Golden, CO, 80401 – USA

1. **Notes**

**Supplementary note 1**

The proposed functionalization-mediated transformation proceeds through four successive steps (**Figure 1a and Figure S1a**). First, sulfur-rich amorphous MoS_x_ clusters are generated from the molybdenum sulfide precursor. Second, cysteine coordinates to the MoS_x_ surface through its sulfur- and nitrogen/oxygen-containing functional groups, creating a ligand-rich local environment. Third, the addition of Fe species yields the MoS_x__Fe_Cyt intermediate, in which cysteine promotes the association of Fe with the sulfur-rich MoS_x_ framework. Finally, thermal treatment at 210 °C induces ligand decomposition and reorganization of the Mo–S framework into MoS_2_ nanosheets. During this conversion, the sulfur-rich coordination environment is proposed to suppress Fe aggregation and favor the formation of bridge-like Fe coordination sites on MoS_2_, as subsequently supported by X-ray absorption spectroscopy and theoretical modelling. In this study, we first synthesized molybdenum sulfide with a high sulfur content (MoSₓ, x>2) through acid reduction using (H_2_SO_4_) coupled with ascorbic acid (AA) as a reducing agent. After the complete evolution of hydrogen sulfide (H_2_S) gas, the resulting compound was initially expected to correspond to MoS_3_ ^[1–3]^. However, Energy-dispersive X-ray spectroscopy (EDS, **Figure S1b**) revealed a Mo:S ratio of 1:3.7. Furthermore, X-ray diffraction (XRD) analysis showed a broad amorphous peak around 14° (**Figure S2a**), confirming the amorphous nature of the synthesized material. Therefore, the data suggest that our synthesized amorphous molybdenum sulfide presents a coordination polymer framework composed of [Mo_3_S_13_]^2−^ entities, where two out of three terminal disulfide bonds are shared ^[4]^. This catalyst displays characteristics compared to those recently documented ^[5–7]^, accordingly, the obtained material will be referred to as MoSₓ in the following sections. After chemical functionalization with Fe_Cyt complex, the new matrix (MoS_x__Fe_Cyt) remains amorphous, marked by the low intensity of the 002 peak at ~14^°^ (**Figure S2a**) ^[8–10]^. In addition, a distinct peak is observed at approximately 18° (* in **Figure S2a**), which may be attributed to the formation of an Fe_Cyt complex; however, the exact origin of this peak remains unclear. The structural evolution of MoS_x_ and MoS_x__Fe_Cyt were further followed by Raman spectroscopy (**Figure S2b**). Vibrations corresponding to bridging/shared disulfide (*ν*_(S–S)br/sh_) and terminal disulfide (*ν*(S–S)_t_) are clearly observed around 555 cm^-1 [4]^. Additionally, molybdenum sulfide bonds are identified at *ν(*Mo–S) frequencies ranging from 380 to 280 cm^-1^, with the *ν*(Mo_3_–μ_3_S) vibration resonating at 450 cm^-1^ ^[5,6]^. After functionalization, a new peak appears around 500 cm^-1^ **(*** in **Figure S2b)**, likely corresponding to the formation of an Fe_Cyt complex. This may also suggest also that MoS_x_ promotes the oxidation of thiol cysteine into cystine ^[11]^, which is then coordinated by iron. This functionalization is further supported by Fourier transform infrared spectroscopy (FTIR) that show the presence of the ligand as well as the characteristic vibration of Fe_Cyt complex (* in **Figure S2c**). **We then evaluated the structure of** MoS_x__Fe_Cyt **using transmission electron microscopy (TEM), which** reveals 2D agglomerated sulfur/molybdenum-rich clusters (**Figure S3a**).

It has been previously shown that the thermal decomposition of the rod-shaped, sulfur/molybdenum-rich precursor at 450–1000 °C acts as a self-templating process, allowing it to retain its morphology while converting into MoS_2_ ^[12]^. However, unlike hydrothermal synthesis, which forms crystals from solution under moderate temperatures and high pressure, our goal is to create a MoS_2_-like structure coordinated with Fe, starting from our sulfur/molybdenum-rich cluster with a high sulfur content. As previously shown, directly mixing sulfur/molybdenum-rich precursor and an Fe salt can produce well-defined Fe-MoS_2_ with phases mixing ^[13]^ or Fe doped MoS_2-x_ ^[14]^. Similarly, mixing an amine with sulfur/molybdenum-rich precursor under acidic conditions at moderate temperature leads to the formation of a 1T/2H MoS_2_ mixture ^[15]^. In addition, previous study has also investigated that the synthesis of a MoS_3_ intermediate can be transformed *via* a hydrothermal process into MoS_2_ ^[16]^. Thus, we aimed for our MoS_x_ to readily reorganize and condense under mild thermal conditions, serving as an ideal sulfur/molybdenum-rich cluster for the formation of MoS_2_-like frameworks. Then, we anticipate that the formation of the MoS_x__Fe_Cyt sulfur/molybdenum-rich precursor facilitates solvothermal synthesis at moderate temperatures, where incomplete rearrangement of the Mo-S clusters leads to local structural disorder that can also help in PEMWE application ^[17]^.

**Supplementary note 2**

***b-*Fe-MoS_2_ was obtained by the solvothermal treatment of** MoS_x__Fe_Cyt sulfur/molybdenum-rich precursor at 210 °C; 18 h. **Following this treatment of** MoS_x__Fe_Cyt, **the XRD patterns reveals a shift of the characteristic (002) diffraction peak to 0.93 nm** (**Figure S3b**). The shifted XRD peaks in ***b-*Fe-MoS_2_** and increased interlayer spacing maybe suggest Fe integration ^[13]^ and improved crystallinity compared to MoS_x__Fe_Cyt. XRD alone cannot determine whether Fe is incorporated into the lattice and coordinated or the observation was due to the intercalation of the Fe_Cyt ligand in MoS_2_. However, it was previously shown that aggregation promoted by [MoS_4_]^2-^ reduction facilitates hydrothermal synthesis and results in the intercalation of molecules, such as aromatic compounds. However, the cysteine is much more easily decomposed compared to aromatic molecules ^[15]^. Additionally, note that low-temperature synthesis also can promotes oxygen incorporation *via* preserved Mo-O bonds, contributing to controlled disorder and increased interlayer spacing ^[18]^. We believe that this shift was associated with a less ordered structure ^[19]^ and the incorporation of Fe, which was subsequently confirmed using more powerful techniques. Raman spectroscopy was employed to further confirm the phase composition of ***b-*Fe-MoS_2_**. As shown in **Figure S3c**, the broadening of the modes at 379 cm^-1^ (E_2_^1^g) and 406.9 cm^-1^ (A_1_g) is primarily associated with the 2 H phase of MoS_2_. To further examine the electronic structure of ***b-*Fe-MoS_2_**, we performed high-resolution Mo 3d XPS measurements and compared the spectrum with that of crystalline CVD-grown 2H-**MoS_2_** (**Figure S3d**) ^[13]^. The CVD-grown 2H-**MoS_2_** reference displays sharp Mo^4+^ components characteristic of a comparatively ordered 2H framework. In contrast, ***b-*Fe-MoS_2_** exhibits substantially broadened Mo 3d features that can be fitted with contributions assigned to lower-binding-energy 1T′-like Mo environments at 229.2 eV (Mo 3d_5/2_) and 232.3 eV (Mo 3d_3/2_), together with 2H-like components at 230.4 eV (Mo 3d_5/2_) and 233.5 eV (Mo 3d_3/2_). This is also confirmed by our high-resolution HRTEM and STEM images, which revealed to traditional zig-zag pattern of the Mo atoms from the 1T’ phase (**Figure S4a-c**). Considered together with the Raman spectroscopy and other structural characterization, these results are consistent with a mixed, defect-rich **MoS_2_** framework containing electronically heterogeneous Mo environments. Relative to the crystalline 2H-**MoS_2_** reference, the broader Mo 3d envelope and increased contribution from lower-binding-energy components in ***b-*Fe-MoS_2_** are consistent with modified local electron density around Mo sites and increased structural disorder associated with the Fe-containing, partially reorganized **MoS_2_** framework. However, it has been previously reported that MoSₓ (2 ≤ x ≤ 3) nanostructures can be synthesized *via* a simple solvothermal method at a low temperature (200 °C), using (NH_4_)_2_MoS_4_ as precursor and DMF as the solvent, while fully crystalline MoS_2_ can be obtained by subsequent annealing at high temperature ^[20]^. XRD, Raman and XPS analyses indicate that our MoS_2_ exhibiting a mixed structure consistent with the temperature of the solvothermal synthesis.

**Supplementary note 3**

The morphology and atomic structure of *b-*Fe-MoS**_2_** were first investigated by TEM, HRTEM, and STEM-EDS. The low-magnification TEM image confirms the formation of flake-like MoS**_2_** nanosheets (**Figure S4a**). HRTEM images reveal clear parallel lattice fringes at the sheet edges, indicating the layered structure of the material. The measured interlayer spacing is approximately **0.94 nm** (**Figure S4b**), which is larger than the typical basal spacing of pristine MoS**_2_**, usually around **0.62 nm for the (002) plane**. This expanded spacing suggests that Fe incorporation enlarge the distance between adjacent MoS**_2_** layers. This expanded spacing is close to that one observed in XRD results. Atomic-resolution STEM-EDS images further confirm the crystalline nature of *b-*Fe-MoS**_2_** and the presence of Fe (**Figures S4c-g**). The observed periodic atomic contrast and Moiré patterns demonstrate lattice ordering within the multi-nanosheets, while STEM–EDS elemental mapping confirms the presence and spatial distribution of Mo, S, and Fe in the *b-*Fe-MoS_2_ nanosheets (**Figures S4c-g**). The Mo and S maps show strong and overlapping signals throughout the analyzed region, confirming the preservation of the MoS_2_ framework. The weak Fe intensity is expected for dopant-level incorporation and suggests that Fe is dispersed rather than forming large Fe-rich aggregates (**Figure S4e-g**). Atomic-resolution STEM imaging was employed to examine the local structure of b-Fe-MoS₂ nanosheets. The overview image reveals extended locally ordered domains within the nanosheet (**Figure 2c**). Magnification of the selected area identifies adjacent bilayer (BL) and single-layer (SL) domains separated by a clearly visible boundary (**Figure 2d**). The single-layer region is particularly useful for structural analysis because it minimizes contrast overlap arising from stacked MoS_2_ layers. The intensity profile extracted along the indicated line in the SL domain shows localized variations in atomic contrast within the ordered MoS_2_ framework (**Figure 2e**). These contrast variations are consistent with local structural/electronic heterogeneity in the ultrathin *b-*Fe-MoS_2_ nanosheets. Therefore, when considered together with the absence of detectable Fe–Fe scattering in the Fe K-edge EXAFS spectrum and the closer agreement of the experimental spectrum with the CN3 bridge-like model than with the CN4 atop-like model, these STEM observations provide complementary support for a dispersed Fe environment with a predominantly bridge-like local coordination in *b-*Fe-MoS_2_. In addition, X-ray photoelectron spectroscopy (XPS) analyses of the Fe 2p region from ***b-*Fe-MoS_2_** can be deconvoluted into two pairs of peaks at 708.1/720.7 eV and 710.05/723.15 eV, attributed to Fe from the Fe-S and satellite peaks, respectively (**Figure 2f)**^[13]^**.** The Fe 2p spectrum suggests the existence of Fe-S bonds without the presence metallic Fe in ***b-*Fe-MoS_2_,** further supporting atomically coordinated Fe on MoS_2_.

**Supplementary note 4**

The GC_alone system (**Figure S7a)** demonstrated low and stable current densities during oxidation/reduction process, the small decrease likely caused by mass transport limitations or bubble accumulation on the electrode surface. Before starting LSV, ***b-*Fe-MoS_2_**-based catalysts are activated using CV (**Figure S7b**). The polarization curves (**Figure S7c**) provided a direct comparison of catalytic activity across systems. GC_alone exhibited negligible activity, with minimal current response under applied potentials. In contrast, ***b-*Fe-MoS_2_**/GC achieved the highest current density, indicative of its excellent HER kinetics. MoS_x_ _Fe_Cyt/GC displayed intermediate performance. These results underscore the superior electron transport properties and active site availability of ***b-*Fe-MoS_2_** catalysts as shown previously in CP support. Nyquist plots from EIS experiments revealed crucial insights into the charge transfer resistance (R_ct_) of the materials. For MoS_x_ _Fe_Cyt/GC (**Figure S7d**), the large semicircles across all potentials indicated significant R_ct_, reflecting poor electron transfer and HER kinetics. ***b-*Fe-MoS_2_**/GC (**Figure S7e**), however, exhibited substantially smaller semicircles, particularly at higher potentials, enhanced conductivity and improved charge transfer properties. These findings suggest that the inclusion of Fe in MoS_2_ structures effectively reduces interfacial resistances, promoting catalytic activity. The durability of ***b-*Fe-MoS_2_/**GC was tested under continuous operation (**Figure S7f**). The potential remained stable with small decrease due to the lower physical attachment of catalyst and bubbles accumulation.

**Supplementary note 5**

To get understanding of the origin of the improvement of the catalytic performance, we examined the role of the configuration of the Fe atoms. The comparison between cysteine-mediated ***b-*Fe-MoS_2_** and our previously synthesized ***a-*Fe-MoS_2_** ^[13]^ shows a difference in the total current density, which is attributed to the configuration of the Fe atom sites. Structural insights of ***b-*Fe-MoS_2_** were extracted from EXAFS fitting and used for DFT calculations. We compared these experimental results to our calculations based on the structural models presented in **Figure 2j-k**. We computed the free energy of hydrogen adsorption ($\Delta G_{H^{\text{*}}}$) to gauge the intrinsic HER properties of the different samples. It should be noted that a perfect MoS_2_ structure was used to simulate this effect, maintaining the same coordination environment as observed in the FT-EXAFS data. ***b-*Fe-MoS_2_** demonstrates lowest energy barrier for HER at 0.21 eV compared to MoS_2_ complexed with Fe_Cyt (used as reference) indicating superior catalytic efficiency **(Figure 3b)**. In contrast, previously synthesized ***a-*Fe-MoS_2_** ^[13]^ show a value of 0.10 eV, indicating high catalytic efficiency as show in LSV results (**Figure 3b**). We then examined the charge-density redistribution in ***b-*Fe-MoS_2_** and **MoS_2_** complexed with Fe-Cyt (**Figure 3c**). The charge-density-difference maps indicate that Fe incorporation modifies the electronic structure of **MoS_2_**, producing regions of electron accumulation and depletion within the Fe-modified framework. The blue regions correspond to electron accumulation, while the green regions indicate electron depletion. Bader charge analysis yields a charge-transfer magnitude of |0.87e| for ***b-*Fe-MoS_2_** slightly lower than that of MoS_2__Fe_Cyt (|0.92e|), demonstrating a smaller extent of electronic redistribution in ***b-*Fe-MoS_2_**. Comparison with our previously synthesized ***a-*Fe-MoS_2_** ^[13]^, which exhibits an even lower charge-transfer magnitude of (|0.79e|), further confirms that the Fe coordination environment strongly influences charge redistribution within the MoS_2_ framework. This result indicates that enhanced HER performance in this system is not associated with maximal charge redistribution at the Fe-containing interface. Instead, the different charge-transfer values evidence distinct local electronic environments of Fe that may influence hydrogen-intermediate binding and catalytic kinetics. LSV measurements with the *in-situ* catalyst revealed that our previously developed ***a-*Fe-MoS_2_** and ***b-*Fe-MoS_2_**-CP samples exhibited comparable performance. In contrast, the spray-coated ***b-*Fe-MoS_2_** displayed a lower current density. We then examined the robustness of the catalysts under continuous HER operations. Compared to MoS_x_, MoS_x__Fe_Cyt and the previously synthesized ***a-*Fe-MoS_2_** ^[13]^, our newly ***b-*Fe-MoS_2_** demonstrated a clear improvement in stability at -10 mA cm^-2^ with lower degradation rate over time (**Figure 3d**). The stability of the ***b-*Fe-MoS_2_** catalyst was evaluated over a period of 70 hours, indicating robust durability (**Figure 3e**). The configuration of Fe atoms in ***b-*Fe-MoS_2_** critically influences its catalytic performance and stability. We show that the cysteine-mediated ***b-*Fe-MoS_2_** shows enhanced HER activity and stability with a coordination number of almost 3. When the Fe atom in ***a-*Fe-MoS_2_** ^[13]^ has a different coordination position, the catalytic performance improves further, though at the expense of stability. These findings highlight the importance of Fe site configuration in optimizing MoS_2_-based catalysts and stability.

**Supplementary note 6**

HRTEM-STEM/EDS mapping before and after HER further confirms that the MoS_2_ framework is largely maintained under electrocatalytic conditions (**Figures S9b-c**). Mo and S remain broadly co-localized after HER, while Fe remains detectably dispersed across the nanosheet. The structural evolution of *b-*Fe-MoS_2_ after HER was then examined by STEM (**Figure S9d**). Before HER (**Figure S4c**), the material shows well-resolved crystalline domains with clear periodic lattice contrast. After HER (**Figure S9d**), the atomic contrast appears more irregular, suggesting electrochemical reaction during HER. This may involve defect generation and sulfur vacancy formation.

**Supplementary note 7**

The performance of ***b-*Fe-MoS_2_** based catalysts for the hydrogen evolution reaction (HER) was evaluated in a custom-made PEMWE cell as describe before ^[21]^ (**Figure 5a**). The catalyst was deposited onto Nafion N115 membranes using a decal strategy, resulting in the fabrication of catalyst-coated membranes (CCMs, **Figures 1b and 5b**). These CCMs were subsequently integrated with an IrOₓ-based anode catalyst and either b-Fe-MoS_2_/C or Pt/C cathode catalysts, the latter serving as the reference material (**Figures 5a and 5d**). The assembled system included a porous transport layer (PTL) and a gas diffusion layer (GDL). The CCMs featured an IrOₓ anode loading of 2.5 mg cm^-2^ with cathode loadings, 2.5 mg cm^-2^ for ***b-*Fe-MoS_2_**/C Vulcan® (50 wt%) or 0.5 mg cm^-2^ for Pt/C (60 wt%). The ***b-*Fe-MoS_2_** loading was selected based on previous studies that demonstrated a performance plateau at this level for similar materials ^[22–24]^. The operational response of the IrOₓ//***b-*Fe-MoS_2_** and IrOₓ//Pt/C CCMs was evaluated using a 160-h dynamic-operation stress protocol at 80 °C. The protocol consisted of repeated 48 h sequences comprising 24 h operation at 1.0 A cm^-2^ followed by 24 h operation at 0.5 A cm^-2^. Within each 24 h segment, approximately 3 h were used for EIS and polarization-curve measurements, while approximately 1 h corresponded to controlled shutdown/restart periods designed to simulate OFF/ON operation. This protocol was selected to probe operational behaviour under dynamic laboratory testing conditions and is not presented as a standardized industrial durability or lifetime-validation procedure. The voltage values used to track performance during the test were extracted at selected current densities from the polarization curves recorded at the indicated times (**Figure 5f**). The cell was operated in a closed-loop configuration with batch water recirculation, enabling up to 24 h of continuous operation and introducing an additional stress associated with the gradual degradation of water quality due to the leaching of different elements from the cell components. Over the 160 hours of ASTs, the IrO_x_//Pt/C system stability was remarkable, with no significant changes in polarization curves (**Figure S10a**). In addition, **Figures S10b-c** shows EIS measurements at varying current densities before and after 160h. Based on this we confirm low and stable high-frequency resistance (HFR) of approximately 105 mΩ cm^2^, without noticeable variation during 160 test period (**Figure S10d**). These results highlight the robustness of the IrO_x_//Pt/C assembly and validate the reproducibility of the CCM fabrication methodology as well as the PEMWE set-up (results supported by HFR-free cell voltage presented in **Figure S11**. We then perform the same 160-hour ASTs using ***b-*Fe-MoS_2_** as cathode catalyst (**Figures 5e**). However, IrO_x_//***b-*Fe-MoS_2_** (without prior initial activation) revealed a significant decrease in cell voltage during the first few hours, followed by stabilization and a continuous improvement over the 160-hour period (**Figure 5e**). Initially, the HFR was approximately twice as high at t=0h compared to t=20h, eventually stabilizing at ~130 mΩ cm^2^ after 160 hours (**Figure S12 and S13a**). The HFR-Free cell voltage at each time was then evaluated at 200 mA cm^-2^ (**Figure S13b**). The initial performance drop and the HFR higher value compared to IrO_x_//Pt/C can be explained by mass transport limitations caused by the increased thickness of the catalytic layer (**Figure S13c**). These effects became negligible after the initial activation phase. The ***b-*Fe-MoS_2_** catalyst exhibited stable HFR values after activation, with no significant changes during the stability test. This stability was also reflected in the HFR-free polarization curves indicating consistent cell performance (**Figures S14**). Collectively these results indicate a highly efficient and stable non-noble metal catalyst for HER.

2. **Materials**

Ammonium tetrathiomolybdate (NH_4_)_2_MoS_4_, Iron(III) nitrate nonahydrate (Fe(NO_3_)_3_,9H_2_O, ≥98%), DMF (ACS reagent), sulfuric acid (H_2_SO_4_, 99.99%), isopropyl alcohol (ACS reagent) and L-cysteine were purchased from Sigma-Aldrich. All the reagents were used as received without further purification. The MoS_2_ bulk powder was purchased from Alfa Aesar. Nafion™ D521CS perfluorinated solution (ion power, 5% weight) was used in this work. Carbon black vulcan (XC 72) and carbon paper (CP, sigracet 39 AA brand: SGL carbon) were obtained from fuel cell store. All chemicals were used as received. All aqueous solutions were prepared using deionized water.

3. **Synthesis of the catalysts**

The synthesis strategy aims to obtain iron-functionalized molybdenum sulfide materials starting from a molecular rich Mo/S cluster. For this purpose, an aqueous solution of high concentration (30 mM of (NH_4_)_2_[MoS_4_]), well-sonicated was kept under an argon atmosphere and stirred for 25 minutes. Subsequently, 1 g of L-ascorbic acid was added to the mixture, resulting in a color change from brown to black. The solution was then continuously stirred under an argon atmosphere for 2 hours at 20 °C. Afterwards, 3 drops of concentrated H_2_SO_4_ were added, and the resulting solid phase (denoted as MoS_x_) was recovered by centrifugation (10 minutes, 4,400 rpm, 20 °C). To introduce iron species, Fe-L-cysteine complex (Fe-Cyt) is prepared and mixed with the MoS_x_ dispersion. Therefore, the MoS_x_ product was then dispersed in 50 mL of isopropyl alcohol (IPA), subjected to sonication for 20 minutes, and kept under argon with stirring for an additional 10 minutes. Subsequently, 10 mL of L-cysteine solution (300mg, H_2_O) was added to the previous suspension, followed by stirring under argon for 10 minutes. Then, 600 mg of Fe(NO_3_)_3_,9H_2_O was introduced into the solution, and the reaction was terminated after 2 hours. The resulting solid product was recovered in 5 mL excess solution, and the rest was removed (MoS_x__Fe_Cyt). After this, the new solution was dispersed in 250 mL dimethylformamide (DMF) and transferred to adopted solvothermal autoclave reactor volume (in our case we use the 25 mL or 150 mL one). The CP (2.5 × 2.5 cm) was then directly added to the solvothermal system, which was heated at 210 °C for 18 h under saturated argon atmosphere, resulting in the sample labeled ***b-*Fe-MoS_2_** (for the powder) and ***b-*Fe-MoS_2_** _CP (for the *in situ* one). The solid product was recovered first *via* filtration and then washed by centrifugation at 10K and 20 °C for 10 minutes using IPA and water. The final coating concentration for ***b-*Fe-MoS_2_**_CP was calculated to be at 1.2 mg cm^-2^. Electrodes of MoS_x_, MoS_x__Fe_Cyt, and ***b-*Fe-MoS_2_** were fabricated using spray technique into CP. The ink was prepared using a mixture of 150 μL of Nafion ionomer and 200mg of catalyst dispersed in 9mL of IPA and 1mL of H_2_O and sonicated for 3h. 30 mg of carbon Vulcan was added to the mixture of ink. The final coating concentration was maintained at 1.2 mg cm^-2^. Electrodes into glassy carbon (GC) were fabricated using drop casting method. The Ink was prepared using a mixture of 20 μL of Nafion and 10 mg of catalyst dispersed in 1mL of IPA and 0.1 mL of H_2_O and sonicated for 1h. Then, 20 μL of prepared solution was drop casted into GC surface. All experimental procedures for catalysts synthesis and electrode preparation, including reaction steps, filtration, and material recovery prior to centrifugation, were performed in a well-ventilated fume hood.

4. **Techniques of characterization**

Transmission electron microscopy (TEM) was performed using a JEOL 1400 flash microscope equipped at 120 kV. X-ray photoelectron spectroscopy (XPS) analyses were conducted on an ESCALAB 250 system (Thermo Electron) using a monochromatic Al Kα line (1486.6 eV) as the excitation source, with an analyzed surface diameter of 500 µm. X-ray diffraction (XRD) patterns were obtained using an X'Pert Powder diffractometer. Fourier-transform infrared (FTIR) spectroscopy was carried out using a Nexus spectrometer (ThermoFisher). Scanning electron microscopy (SEM) was conducted on a Hitachi S4800, while SEM-EDX analysis was performed using a Zeiss EVO HD15 microscope equipped with an Oxford Instruments X-Max N SDD EDX detector. Raman spectra (RS) were recorded using a Renishaw inVia Raman microscope with a 532 nm excitation wavelength and a ×50 objective lens. HRTEM images were acquired using a Titan G2 60-300 microscope (FEI) equipped with an X-FEG emission gun, operating at an acceleration voltage of 300 kV. High-angle annular dark-field (HAADF) scanning transmission electron microscopy (STEM) analysis was carried out on another Titan G2 60-300 microscope (FEI) integrated with a HAADF 3000 detector (Fishione). Elemental mapping was performed via energy-dispersive spectrometry (EDS) in STEM mode using the Super-X system, which incorporates four silicon drift detectors (Bruker), with an acquisition duration of 20 min. Fe K-edge X-ray absorption fine structure (XAFS) measurements were conducted at the BL14W1 beamline of the Shanghai Synchrotron Radiation Facility (SSRF, Shanghai, China) with Si (111) crystal monochromator. Prior to analysis, samples were pressed into thin sheets with 1 cm in diameter and sealed with Kapton tape. The XAFS spectra were collected at room temperature using a 4-channel Silicon Drift Detector (SDD) Bruker 5040. Fe K-edge extended X-ray absorption fine structure (EXAFS) spectra were recorded in transmission mode. Negligible changes in the line-shape and peak position of Fe K-edge XANES spectra were observed between two scans taken for a specific sample. Reference standards, including Fe foil, Fe_2_O_3_, and FeS, were also measured in transmission mode for comparative analysis. XAS data processing was performed using Athena, and EXAFS fitting was carried out using Artemis^[25]^. The CIF file of the CN3 structure used for the DFT calculations was employed to generate the Fe–S and Fe–Mo scattering paths for fitting, while the Fe–O scattering path was obtained from the Fe_2_O_3_ CIF structure. The S_0_^2^ value of 0.82 was determined by fitting the Fe foil reference measured during the sample data acquisition.

5. **In-Situ high-energy X-ray diffraction (HE-XRD) using a capillary electrochemical Cell**

Electrochemical cell with a capillary working electrode was designed for in situ X-ray diffraction measurements. The in-situ HE-XRD experiments were carried out at the Deutsches Elektronen-Synchrotron at room temperature with a homemade cell. The as-prepared catalysts containing amorphous carbon powder support to simulate our real reaction (in three electrodes) were loaded in a carbon capillary and used as the working electrode, along with graphite as the counter electrode and an Ag/AgCl electrode as the reference electrode. The electrolyte solution 0.5M H_2_SO_4_ was circulated throughout the cell. The ep­oxy-aligned carbon fiber capillary [working electrode](https://goldbook.iupac.org/W06686.html) is connected to the electrolyte reservoir containing the counter electrode, and [reference electrode.](https://goldbook.iupac.org/R05229.html) Electrolyte is recirculated via peristaltic pump to replenish the electrolyte species. Detailed settings of the cell have been described in previous work^[26]^. The working electrodes were pre-treated by full cyclic voltammetry to stabilize the catalyst surface. The obtained HE-XRD data was converted to PDF by using PDFgetX3^[27]^. The HE-XRD experimental data was first reduced to the structure factors, S(q), and then Fourier transferred to atomic pair distribution functions G(r).

6. ***In situ* Raman**

The cell consists of a compact (**Figure S8c**), layered assembly enabling simultaneous electrochemical control and optical access. The working electrode is positioned within an insulating holder and electrically contacted via conductive leads. A sealing O-ring defines the electrolyte chamber and prevents leakage. The top part of the cell incorporates a quartz window aligned with the electrode surface, allowing the Raman laser to be focused directly onto the electrode–electrolyte interface. Electrical feedthroughs integrated into the cell body enable connection to the potentiostat while maintaining a sealed environment. This design ensures stable alignment, minimal optical losses, and compatibility with standard microscope stages for in situ measurements. Raman measurements were conducted using a Renishaw in Via Raman microscope and an ×50 objective (Leica) equipped with a 532 nm laser. The spectra were recorded using the Renishaw WiRE software (version 4.4). An Ag/AgCl electrode and a Pt plate were used as the reference and counter electrodes respectively controlled by PalmSens EmStat4S.

7. **Electrochemical characterization in a three-electrode cell**

The electrodes were prepared using a spray-coating deposition strategy on carbon paper (CP), solvothermal *in situ* method or drop casting into glassy carbon. The electrodes were 10 times cycled between 0 and -0.3 V *vs.* RHE at 10 mV s^-1^. Following the initial stabilization of the electrode, the catalytic properties were evaluated. All electrochemical measurements were conducted using the electrochemical workstation (VSP 300 potentiostat Bio-Logic Science Instruments and PARSTAT MC Multichannel Potentiostat, PMC-200). Electrochemical experiments were conducted in a three-electrode cell setup with saturated calomel electrode (SCE) as the reference electrode and graphite as the counter electrodes. The electrolyte (0.5M H_2_SO_4_) was used in this work. Linear sweep voltammetry (LSV) was recorded then the current density was calculated using the surface area of the original used electrode. All the potentials in our work were calculated relatively to RHE, according to the Nernst equation:

E (V *vs.* RHE) = E (V *vs.* SCE) + 0.059pH + *E*^°^_SCE_

Long-term stability was tested using Chronopotentiometry (v-t). The 12 h stability dataset was used to calculate the degradation rate, while long-term chronopotentiometry was conducted for 70 h, Raman monitoring was performed for 5 h, and *ex situ* XPS analysis was carried out between 0 and 100 h of HER operation. Electrochemical impedance spectroscopy (EIS) test was performed within the frequency range of 100 kHz-0.01 Hz with constant potential amplitude.

8. **Preparation of membrane electrode assemblies and electrochemical characterization in a single-Cell PEM electrolyzer**

The PEMWE cell used in our experiments is a custom design. The anode plate is made from Grade 1 titanium and includes 25 parallel straight channels, each 1 mm deep and separated by 1 mm. The flow area of the anode plate is 5x5 cm^2^ (**Figure 5a-c**). The cathode plate, fabricated from 316L stainless steel, is coated with a 1 μm layer of wood nickel followed by a 1.6 μm layer of gold. These coatings ensure optimal contact with the 325 μm-thick Toray 120 GDL. Like the cathode, the anode plate also features 25 straight parallel channels. To accommodate thermal expansion during operation, the anode and cathode plates are held together by a compression system consisting of multi springs. For the full-cell characterization of ***b-*Fe-MoS_2_**, a catalyst ink spray-coating method was employed to fabricate catalyst-coated membranes (CCMs). Sono-Tek ExactaCoat inkjet system was used for the deposition of the catalysts directly onto PTFE inert substrate and subsequently transferred onto Nafion™ 115 membranes via the so-called decal transfer to form catalyst-coated membranes (CCMs) ^[28]^. The cathodic catalyst layer consisted of 2.5 mg cm^-2^ of ***b-*Fe-MoS_2_** (50 wt%) or 0.5 mg cm^-2^ Pt/C (60 wt%) as a reference. On the anode side, custom-made anodic CCMs coated with 2.5 mg cm^-2^ of IrO_x_ catalyst were used (Surepure Chemetals). The cathodic part was integrated with a gas diffusion layer (Toray 120 carbon paper, 370 µm, 5% Wet Proofing, fuel cell store) and a titanium porous transport layer (PTL) was used on the anode side (Sintered Ti Gr. 1, 1mm thick, 40% porosity, Powder size ~75 µm, size pores of 10 µm).

The catalyst ink for ***b-*Fe-MoS_2_** (50 wt% Vulcan® XC 72) was prepared by dispersing 100 mg of catalyst with 250 mg of Nafion™ solution (20 wt%), 15 mL of deionized water, and 15 mL of isopropanol. The mixture was ultrasonicated for 9 h to ensure homogeneous dispersion.

Electrochemical characterization was conducted under operating conditions of 80 °C with alternating current densities over a 160-hour (AST) or 360 h stability test. Electrochemical performance was assessed through polarization curves, as well as through electrochemical impedance spectroscopy (EIS) from 100 kHz to 0.1 Hz at various current densities, using an electrochemical workstation (VSP 300 potentiostat Bio-Logic Science Instruments). All experiments were conducted under a total clamping force of 15 kN. Ultrapure water with a resistivity of 18.2 MΩ cm at 25 °C was used throughout the tests. The theoretical thermoneutral voltage for PEM at 80 °C, namely 1.42 V ^[29]^. In this work, the energy efficiency of the electrolysis cell is defined as the ratio between the higher heating value (HHV) of the produced hydrogen and the electrical energy consumed during electrolysis. According to this definition, a cell operating at 1.47 V at 1 A cm^-2^ reaches 100% efficiency ^[30]^.

9. **Computational details**

All DFT calculations were implemented in the Vienna Ab-initio Simulation Package (VASP) code with a projector augmented-wave (PAW) method. The exchange-correction energy was described using a generalized gradient approximation (GGA) with a Perdew–Burke–Ernzerhof (PBE) format. A plane-wave basis with a kinetic energy cutoff of 520 eV was chosen to expand the electronic wave functions. We chose a 5×5×1 supercell of monolayer MoS_2_ with a 20 Å vacuum layer above the basal plane and then placed one Fe atom and a Cys molecule on the Mo top. All the structures were allowed to fully relax to the ground state with the convergence of the energy and the forces to 1.0 × 10^-5^ eV and 0.01 eV Å^-1^. A 3 × 3 × 1 Γ-centered Monkhorst-Pack schemed k-mesh was used to sample the first Brillouin zone. The DFT-D2 of Grimme was used for the long-range dispersion correction.

**Supplementary Figure S1. (a)** Schematic illustration of the functionalization-mediated transformation from MoSₓ clusters to bridge-coordinated *b*-Fe-MoS_2_ and (**b)** SEM-EDS analysis of prepared MoS_x_

**Supplementary Figure S2. (a)** XRD patterns; (**b)** Raman spectra and **(c)** FTIR spectra of prepared MoS_x_ and MoS_x__Fe_Cyt. (*) asterisks are explained in the text.

**Supplementary Figure S3.** **(a)** TEM images of MoS_x__Fe_Cyt; **(b)** XRD patterns comparing ***b-*Fe-MoS_2_** and MoS_x__Fe_Cyt; **(c)** Raman spectra of ***b-*Fe-MoS_2_** and **(d)** Mo 3d high resolution XPS spectra of ***b-*Fe-MoS_2_** compared to CVD-MoS_2_

**Supplementary Figure S4. (a)** TEM; **(b)** HRTEM, (**c-d)** STEM and **(e-g)** STEM-EDS images of ***b-*Fe-MoS_2_** showing the presence of Fe and zigzag patterns of the Mo atoms from.

the 1T’ phase and MoS_2_ patterns from the multilayer regions.

**Supplementary Figure S5.** Fe K-edge XANES analysis of Fe oxidation states in b-Fe-MoS_2_ and a-Fe–MoS_2_

**Supplementary Figure S6.** Scheme electrode preparation using spray technique and SEM-EDS mapping analysis of (**a)** MoS_x__Fe_Cyt and **(b) *b-*Fe-MoS_2_**

**Supplementary Figure S7.** **(a)** Activation of GC alone using cyclic voltammetry technique; **(b)** Activation of MoS_x_ _Fe_Cyt/GC and ***b-*Fe-MoS_2_** /GC using cyclic voltammetry; **(c)** LSV of activated MoS_x__ Fe_Cyt/GC and ***b-*Fe-MoS_2_** /GC electrodes; **(d)** Nyquist plots of MoS_x__ Fe_Cyt; **(e)** Nyquist plots of ***b-*Fe-MoS_2_** /GC and **(f)** stability of ***b-*Fe-MoS_2_** /GC electrode (at -10 mA cm^-2^). In all analysis rod graphite was used as counter electrode and the electrolyte was N_2_ saturated 0.5M H_2_SO_4_ solutions. **(g)** Tafel slope comparing the *in-situ* electrode and sprayed powder electrode into CP using *b-*Fe-MoS_2_ catalyst. **(h)** Nyquist plots of spray-coated *b*-Fe-MoS_2_ and *in situ*-grown *b*-Fe-MoS_2_-CP electrodes recorded at -0.40 V *vs.* RHE (with similar surface area). **(i)** SEM images show the uniform growth of MoS_2_ nanosheets on the CP fibers with elemental mapping and **(j)** EDS analysis confirm the homogeneous distribution of Mo, S, and Fe; (**k)** TEM image reveal ultrathin layered nanosheets with expanded interlayer spacing (~0.93 nm).

**Supplementary Figure S8.** *Operando* large scale PDFs derived from HE-XRD analysis of **(a)** MoS_x__Fe_Cyt, **(b) *b-*Fe-MoS_2_** under electrochemical condition at the indicted potentials versus RHE and **(c)** *in situ* Raman set-up used in this work

**Supplementary Figure S9. (a)** *Ex situ* XPS as function of HER electrolysis time (using the spray powder of *b*-Fe-MoS_2_) and HRTEM-EDS of *b*-Fe-MoS_2_ (**b)** before and (**c)** after HER; **(d)** STEM images of *b*-Fe-MoS_2_ after HER.

**Supplementary Figure S10. (a)** *i-v* curves of IrO_x_//Pt/C homemade CCM in PEMWE before and after 160h; **(b)** Nyquist plots of IrO_x_//Pt/C at 0h and **(c)** Nyquist plots of IrO_x_//Pt/C at 160h. **(d)** HFR variation under constant current densities of IrO_x_//Pt/C at 0h (black) and 160h (pink)

**Supplementary Figure S11.** HFR-free *i-v* curves of IrO_x_//Pt/C homemade CCM in PEMWE before and after 160h

**Supplementary Figure S12.** Nyquist plots of IrO_x_//***b-*Fe-MoS_2_** homemade CCM in PEMWE at different times

**Supplementary Figure S13.** **(a)** HFR of IrO_x_//***b-*Fe-MoS_2_** homemade CCM in PEMWE at different current density; **(b)** HFR as a function of time at 200 mA cm^-2^ and **(c)** Nyquist plots of IrO_x_//***b-*Fe-MoS_2_** CCM at 200 mA cm^-2^ at different times

**Supplementary Figure S14.** HFR-free *i-v* curves of IrO_x_//***b-*Fe-MoS_2_** homemade CCM in PEMWE as a function of time

**Supplementary Figure S15**. 360h stability test of our PEMWE (80°C) at 400 mA cm^-2^

**Table S1.** EXAFS fitting parameters for the *b*-Fe-MoS₂ catalyst at the Fe K-edge based on the CN3 bridging coordination model.

| Fe-O Coordination Number | 1.32±0.31 |
| --- | --- |
| Fe-O Coordination Length | 1.91±0.02 |
| Fe-O Debye Waller Factor | 0.0033 |
| Fe-S Coordination Number | 2.60±0.22 |
| Fe-S Coordination Length | 2.07±0.01 |
| Fe-S Debye Waller Factor | 0.0033 |
| Fe-Mo Coordination Number | 0.49±0.22 |
| Fe-Mo Coordination Length | 2.07±0.03 |
| Fe-Mo Debye Waller Factor | 0.0033 |
| E0 | 1.72 |
| R Factor | 0.016 |

**Table S2.** Comparison of *b-*Fe-MoS_2_ with PGM and PGM-Free catalysts

Comparison with PGM catalysts:

|  | V_cell_ at J = 1A cm^-2^ | EE%(HHV) | Anode laoding (mg cm^-2^) | Cathode laoding (mg cm^-2^) | Degradation rate (V h^-1^) | Size  (cm^2^) | Ref. | Ref. in **Figs. 6a, 6c** |
| --- | --- | --- | --- | --- | --- | --- | --- | --- |
| TaB_2_-IrO_2_**//Pt/C** | ~ 1.67 | ~ 88 | 0.15 mg_Ir_ cm^-2^ | 0.27 mg_Pt_ cm^-2^ | ~ 0.0003 | 5 | ^[31]^ | Ref 1 |
| IrO_2_**//Pt/C** | ~ 1.78 | ~ 82 | 0.2 mg_Ir_ cm^-2^ | 0.27 mg_Pt_ cm^-2^ | ~ 0.03 | 5 | ^[31]^ | Ref 2 |
| IrO_2_**//Pt/C** | ~ 1.70 | ~ 86 | 0.25 mg_Ir_ cm^-2^ | 1 mg_Pt_ cm^-2^ | ~ 0.012 | 4 | ^[32]^ | Ref 3 |
| IrO_x_**//Pt/C** | ~ 1.68 | ~ 87 | 2 | 0.5 | ~ 0.0003 | 6.25 | ^[33]^ | Ref 4 |
| Ir/Nb_2_O_5-x_**//Pt/C** | ~ 1.58 | ~ 93 | 3 | 1 | ~ 0.0001 | 3.4 | ^[34]^ | Ref 5 |
| **IrO_x_//Pt/C** | 1.63 | 90 | 2.5 | 0.5 | 0.00001 | 25 | Our work | |
| **IrO_x_//*b-*Fe-MoS_2_** | 1.90 | 77 | 2.5 | 2.5 | 0.00001 | 25 |  |  |

Comparison with PGM-Free catalysts cathodes:

|  | V_cell_ at J = 1A cm^-2^ | EE%(HHV) | J (A cm^-2^) at V = 2 | Anode laoding  (mg cm^-2^) | Cathode laoding  (mg cm^-2^) | Size  (cm^2^) | Ref. | Ref. in **Figs. 6b, 6c** |
| --- | --- | --- | --- | --- | --- | --- | --- | --- |
| IrO_2_//[Mo_3_S_13_]^2−^/N doped Carbon**//**Pt/C | ~ 1.93 | ~ 76 | ~1.5 | 1.5 | 3 | 5 | ^[23]^ | Ref 6 |
| IrO_2_//Defect-rich 1T-2H MoS_2_ NS/CFP | ~ 1.95 | ~ 75 | ~1.12 | 3 | 0.14 | 5 | ^[35]^ | Ref 7 |
| IrO_2_//MoS_2_/CFP | ~ 2.10 | ~ 70 | ~ 0.75 | 3 | 3 | 5 | ^[35]^ | Ref 8 |
| IrO_2_// e-MoS_2_ | ~ 2.30 | ~ 63 | ~ 0.3 | 2.5 | 4.5 | 3 | ^[36]^ | Ref 9 |
| IrO_2_//1T-MoS_2_ | ~ 2.00 | ~ 73 | ~ 0.75 | 4 | 1.2 | 5 | ^[37]^ | Ref 10 |
| IrO_2_//MoS_2_/c | <1.80 | 82 | ~ 3.1 | 2 ± 0.1 | 2.4 ± 0.1 | 5 | ^[17]^ | Ref 11 |
| **IrO_x_//*b-*Fe-MoS_2_** | 1.90 | 77 | 1.55 | 2.5 | 2.5 | 25 | Our work | |

**References**

[1]	U. Chang, J. T. Lee, J.-M. Yun, B. Lee, S. W. Lee, H.-I. Joh, K. Eom, T. F. Fuller, “In Situ Self-Formed Nanosheet MoS 3 /Reduced Graphene Oxide Material Showing Superior Performance as a Lithium-Ion Battery Cathode” *ACS Nano* **2018**, acsnano.8b07191.

[2]	Y. Zhou, Y. Li, Q. Wang, Q. Wang, R. Du, M. Zhang, X. Sun, X. Zhang, L. Kang, F. Jiang, “Ultrasmall MoS 3 Loaded GO Nanocomposites as High‐Rate and Long‐Cycle‐Life Anode Materials for Lithium‐ and Sodium‐Ion Batteries” *ChemElectroChem* **2019**, *6*, 3113–3119.

[3]	H. Chen, G. Ke, X. Wu, W. Li, Y. Li, H. Mi, L. Sun, Q. Zhang, C. He, X. Ren, “Amorphous MoS3 decoration on 2D functionalized MXene as a bifunctional electrode for stable and robust lithium storage” *Chemical Engineering Journal* **2021**, *406*, 126775.

[4]	T. Daeneke, N. Dahr, P. Atkin, R. M. Clark, C. J. Harrison, R. Brkljača, N. Pillai, B. Y. Zhang, A. Zavabeti, S. J. Ippolito, K. J. Berean, J. Z. Ou, M. S. Strano, K. Kalantar-zadeh, “Surface Water Dependent Properties of Sulfur-Rich Molybdenum Sulfides: Electrolyteless Gas Phase Water Splitting” *ACS Nano* **2017**, *11*, 6782–6794.

[5]	D. Escalera-López, C. Iffelsberger, M. Zlatar, K. Novčić, N. Maselj, C. Van Pham, P. Jovanovič, N. Hodnik, S. Thiele, M. Pumera, S. Cherevko, “Allotrope-dependent activity-stability relationships of molybdenum sulfide hydrogen evolution electrocatalysts” *Nat. Commun.* **2024**, *15*, 3601.

[6]	P. D. Tran, T. V. Tran, M. Orio, S. Torelli, Q. D. Truong, K. Nayuki, Y. Sasaki, S. Y. Chiam, R. Yi, I. Honma, J. Barber, V. Artero, “Coordination polymer structure and revisited hydrogen evolution catalytic mechanism for amorphous molybdenum sulfide” *Nat. Mater.* **2016**, *15*, 640–646.

[7]	W. Hu, L. Xie, C. Gu, W. Zheng, Y. Tu, H. Yu, B. Huang, L. Wang, “The nature of active sites of molybdenum sulfide-based catalysts for hydrogen evolution reaction” *Coord. Chem. Rev.* **2024**, *506*, 215715.

[8]	Y. Li, M. Fan, B. Yu, C. Wang, X. Yu, J. Ding, G. Qin, L. Yan, K. Yin, L. Wang, “Amorphous molybdenum sulfide nanosheets composed of [Mo3S13]2- active-site motifs for enhancing conversion of Fe3+/Fe2+ in Fenton reaction under neutral condition” *Chemical Engineering Journal* **2024**, *495*, 153463.

[9]	E. Ronge, S. Hildebrandt, M.-L. Grutza, H. Klein, P. Kurz, C. Jooss, “Structure of Nanocrystalline, Partially Disordered MoS2+δ Derived from HRTEM—An Abundant Material for Efficient HER Catalysis” *Catalysts* **2020**, *10*, 856.

[10]	D. Wang, H. Li, N. Du, W. Hou, “Amorphous molybdenum sulfide monolayer nanosheets for highly efficient electrocatalytic hydrogen evolution” *Chemical Engineering Journal* **2020**, *398*, 125685.

[11]	X. Chen, N. C. Berner, C. Backes, G. S. Duesberg, A. R. McDonald, “Functionalization of Two‐Dimensional MoS 2 : On the Reaction Between MoS 2 and Organic Thiols” *Angewandte Chemie* **2016**, *128*, 5897–5902.

[12]	S. M. Islam, J. D. Cain, F. Shi, Y. He, L. Peng, A. Banerjee, K. S. Subrahmanyam, Y. Li, S. Ma, V. P. Dravid, M. Grayson, M. G. Kanatzidis, “Conversion of Single Crystal (NH 4 ) 2 Mo 3 S 13 ·H 2 O to Isomorphic Pseudocrystals of MoS 2 Nanoparticles” *Chemistry of Materials* **2018**, *30*, 3847–3853.

[13]	J. Li, Y. Zhang, C. Liu, L. Zheng, E. Petit, K. Qi, Y. Zhang, H. Wu, W. Wang, A. Tiberj, X. Wang, M. Chhowalla, L. Lajaunie, R. Yu, D. Voiry, “3.4% Solar‐to‐Ammonia Efficiency from Nitrate Using Fe Single Atomic Catalyst Supported on MoS 2 Nanosheets” *Adv. Funct. Mater.* **2022**, *32*, DOI 10.1002/adfm.202108316.

[14]	K. Chen, J. Wang, J. Kang, X. Lu, X. Zhao, K. Chu, “Atomically Fe-doped MoS2−x with Fe-Mo dual sites for efficient electrocatalytic NO reduction to NH3” *Appl. Catal. B* **2023**, *324*, 122241.

[15]	Z. Bian, K. Kato, T. Ogoshi, Z. Cui, B. Sa, Y. Tsutsui, S. Seki, M. Suda, “Hybrid Chiral MoS 2 Layers for Spin‐Polarized Charge Transport and Spin‐Dependent Electrocatalytic Applications” *Advanced Science* **2022**, *9*, DOI 10.1002/advs.202201063.

[16]	S. Duraisamy, A. Ganguly, P. K. Sharma, J. Benson, J. Davis, P. Papakonstantinou, “One-Step Hydrothermal Synthesis of Phase-Engineered MoS 2 /MoO 3 Electrocatalysts for Hydrogen Evolution Reaction” *ACS Appl. Nano Mater.* **2021**, *4*, 2642–2656.

[17]	F. Giacobello, V. Cicciò, M. A. Mancuso, A. Muscolino, S. Siracusano, A. S. Aricò, “Durability of nanostructured non-precious MoS2-based electrocatalysts for H2 evolution in PEM water electrolysis” *J. Power Sources* **2025**, *658*, 238223.

[18]	J. Xie, J. Zhang, S. Li, F. Grote, X. Zhang, H. Zhang, R. Wang, Y. Lei, B. Pan, Y. Xie, “Controllable Disorder Engineering in Oxygen-Incorporated MoS 2 Ultrathin Nanosheets for Efficient Hydrogen Evolution” *J. Am. Chem. Soc.* **2013**, *135*, 17881–17888.

[19]	L. Li, Z. Qin, L. Ries, S. Hong, T. Michel, J. Yang, C. Salameh, M. Bechelany, P. Miele, D. Kaplan, M. Chhowalla, D. Voiry, “Role of Sulfur Vacancies and Undercoordinated Mo Regions in MoS 2 Nanosheets toward the Evolution of Hydrogen” *ACS Nano* **2019**, *13*, 6824–6834.

[20]	Y. Shi, Y. Wang, J. I. Wong, A. Y. S. Tan, C.-L. Hsu, L.-J. Li, Y.-C. Lu, H. Y. Yang, “Self-assembly of hierarchical MoSx/CNT nanocomposites (2<x<3): towards high performance anode materials for lithium ion batteries” *Sci. Rep.* **2013**, *3*, 2169.

[21]	T. Srour, K. Kumar, V. Martin, L. Dubau, F. Maillard, B. Gilles, J. Dillet, S. Didierjean, B. Amoury, T. D. Le, G. Maranzana, “On the contact resistance between the anode and the porous transport layer in a proton exchange membrane water electrolyzer” *Int. J. Hydrogen Energy* **2024**, *58*, 351–361.

[22]	T. Corrales-Sánchez, J. Ampurdanés, A. Urakawa, “MoS2-based materials as alternative cathode catalyst for PEM electrolysis” *Int. J. Hydrogen Energy* **2014**, *39*, 20837–20843.

[23]	P. K. R. Holzapfel, M. Bühler, D. Escalera‐López, M. Bierling, F. D. Speck, K. J. J. Mayrhofer, S. Cherevko, C. V. Pham, S. Thiele, “Fabrication of a Robust PEM Water Electrolyzer Based on Non‐Noble Metal Cathode Catalyst: [Mo 3 S 13 ] 2− Clusters Anchored to N‐Doped Carbon Nanotubes” *Small* **2020**, *16*, DOI 10.1002/smll.202003161.

[24]	J. W. D. Ng, T. R. Hellstern, J. Kibsgaard, A. C. Hinckley, J. D. Benck, T. F. Jaramillo, “Polymer Electrolyte Membrane Electrolyzers Utilizing Non‐precious Mo‐based Hydrogen Evolution Catalysts” *ChemSusChem* **2015**, *8*, 3512–3519.

[25]	B. Ravel, M. Newville, “ATHENA , ARTEMIS , HEPHAESTUS : data analysis for X-ray absorption spectroscopy using IFEFFIT” *J. Synchrotron Radiat.* **2005**, *12*, 537–541.

[26]	M. J. Young, N. M. Bedford, N. Jiang, D. Lin, L. Dai, “In situ electrochemical high-energy X-ray diffraction using a capillary working electrode cell geometry” *J. Synchrotron Radiat.* **2017**, *24*, 787–795.

[27]	P. Juhás, T. Davis, C. L. Farrow, S. J. L. Billinge, “*PDFgetX3* : a rapid and highly automatable program for processing powder diffraction data into total scattering pair distribution functions” *J. Appl. Crystallogr.* **2013**, *46*, 560–566.

[28]	X. Liang, G. Pan, L. Xu, J. Wang, “A modified decal method for preparing the membrane electrode assembly of proton exchange membrane fuel cells” *Fuel* **2015**, *139*, 393–400.

[29]	D. Wang, F. Lin, H. Luo, J. Zhou, W. Zhang, L. Li, Y. Wei, Q. Zhang, L. Gu, Y. Wang, M. Luo, F. Lv, S. Guo, “Ir-O-Mn embedded in porous nanosheets enhances charge transfer in low-iridium PEM electrolyzers” *Nat. Commun.* **2025**, *16*, 181.

[30]	L. Wan, M. Pang, J. Le, Z. Xu, H. Zhou, Q. Xu, B. Wang, “Oriented intergrowth of the catalyst layer in membrane electrode assembly for alkaline water electrolysis” *Nat. Commun.* **2022**, *13*, 7956.

[31]	Y. Wang, M. Zhang, Z. Kang, L. Shi, Y. Shen, B. Tian, Y. Zou, H. Chen, X. Zou, “Nano-metal diborides-supported anode catalyst with strongly coupled TaOx/IrO2 catalytic layer for low-iridium-loading proton exchange membrane electrolyzer” *Nat. Commun.* **2023**, *14*, 5119.

[32]	Y. Park, H. Y. Jang, T. K. Lee, T. Kim, D. Kim, D. Kim, H. Baik, J. Choi, T. Kwon, S. J. Yoo, S. Back, K. Lee, “Atomic-level Ru-Ir mixing in rutile-type (RuIr)O2 for efficient and durable oxygen evolution catalysis” *Nat. Commun.* **2025**, *16*, 579.

[33]	M. Faustini, M. Giraud, D. Jones, J. Rozière, M. Dupont, T. R. Porter, S. Nowak, M. Bahri, O. Ersen, C. Sanchez, C. Boissière, C. Tard, J. Peron, “Hierarchically Structured Ultraporous Iridium‐Based Materials: A Novel Catalyst Architecture for Proton Exchange Membrane Water Electrolyzers” *Adv. Energy Mater.* **2019**, *9*, DOI 10.1002/aenm.201802136.

[34]	Z. Shi, J. Li, J. Jiang, Y. Wang, X. Wang, Y. Li, L. Yang, Y. Chu, J. Bai, J. Yang, J. Ni, Y. Wang, L. Zhang, Z. Jiang, C. Liu, J. Ge, W. Xing, “Enhanced Acidic Water Oxidation by Dynamic Migration of Oxygen Species at the Ir/Nb 2 O 5− x Catalyst/Support Interfaces” *Angew. Chem. Int. Ed.* **2022**, *61*, DOI 10.1002/anie.202212341.

[35]	Z. Xie, S. Yu, X. Ma, K. Li, L. Ding, W. Wang, D. A. Cullen, H. M. Meyer, H. Yu, J. Tong, Z. Wu, F.-Y. Zhang, “MoS2 nanosheet integrated electrodes with engineered 1T-2H phases and defects for efficient hydrogen production in practical PEM electrolysis” *Appl. Catal. B* **2022**, *313*, 121458.

[36]	J. Mo, S. Wu, T. H. M. Lau, R. Kato, K. Suenaga, T.-S. Wu, Y.-L. Soo, J. S. Foord, S. C. E. Tsang, “Transition metal atom–doped monolayer MoS2 in a proton-exchange membrane electrolyzer” *Mater. Today Adv.* **2020**, *6*, 100020.

[37]	A. Piñeiro García, D. Perivoliotis, X. Wu, E. Gracia-Espino, “Benchmarking Molybdenum-Based Materials as Cathode Electrocatalysts for Proton Exchange Membrane Water Electrolysis: Can These Compete with Pt?” *ACS Sustain. Chem. Eng.* **2023**, *11*, 7641–7654.
